# Behavioural Study of the Force Control Loop Used in a Collaborative Robot for Sanding Materials

**DOI:** 10.3390/ma14010067

**Published:** 2020-12-25

**Authors:** Rodrigo Pérez Ubeda, Santiago C. Gutiérrez Rubert, Ranko Zotovic Stanisic, Ángel Perles Ivars

**Affiliations:** 1 Department of Mechanical and Materials Engineering, Universitat Politècnica de València, 46022 Valencia, Spain; scgutier@mcm.upv.es; 2 Institute of Industrial Control Systems and Computing, Universitat Politècnica de València, 46022 Valencia, Spain; rzotovic@isa.upv.es; 3 ITACA Institute, Universitat Politècnica de València, 46022 Valencia, Spain; aperles@disca.upv.es

**Keywords:** robot sanding, robot finishing, inner/outer control loop, force control, collaborative robot

## Abstract

The rise of collaborative robots urges the consideration of them for different industrial tasks such as sanding. In this context, the purpose of this article is to demonstrate the feasibility of using collaborative robots in processing operations, such as orbital sanding. For the demonstration, the tools and working conditions have been adjusted to the capacity of the robot. Materials with different characteristics have been selected, such as aluminium, steel, brass, wood, and plastic. An inner/outer control loop strategy has been used, complementing the robot’s motion control with an outer force control loop. After carrying out an explanatory design of experiments, it was observed that it is possible to perform the operation in all materials, without destabilising the control, with a mean force error of 0.32%. Compared with industrial robots, collaborative ones can perform the same sanding task with similar results. An important outcome is that unlike what might be thought, an increase in the applied force does not guarantee a better finish. In fact, an increase in the feed rate does not produce significant variation in the finish—less than 0.02 µm; therefore, the process is in a “saturation state” and it is possible to increase the feed rate to increase productivity.

## 1. Introduction

Surface finishing operations such as polishing and sanding play an important role within industry. These operations are not only performed with an aesthetic purpose but also for functional reasons. The main objective is to obtain a specified surface roughness. On the other hand, the main drawback of these operations is that they have been commonly carried out manually, which makes them expensive and dependent on operator skill. They are time-consuming and prone to errors [1,2]. In manual operations, the time for polishing pieces represents up to 50% of the total production and costs can reach 15% of the total amount. Therefore, improvements in time efficiency and surface quality are the main objectives for this process [3].

Industrial robots have appeared as an alternative to operators, since, due to their competitive cost, flexibility, programmability, and a large volume of work, they are potentially better suited to automate finishing operations [4,5].

In the last decade, collaborative robots (cobots) have gained popularity within the industry because, in addition to the advantages already mentioned, these robots allow for safe work in conjunction with a human operator [6]. Cobots are an important part in the physical systems of the Smart Manufacturing Systems of Industry 4.0 [7]. These robots are commonly used in applications such as assembly, pick and place, inspection, and welding operations, among others [8]. Research in [9] shows an initial focus on the use of collaborative robots in mould polishing, where automatic polishing by the robot without force control is performed in parallel with manual operation. Polishing a flat surface, the authors demonstrated that a cobot presents a similar result that a 3-axis CNC machine (Hardinge Corporate Headquarters, Westlakes, Berwyn, PA, USA).

In the case of surface finishing applications, it is crucial to control the necessary contact force to ensure the same quality throughout the treated part. To solve this problem, some studies offer several solutions for industrial robots (non-collaborative), where the force control is accomplished passively through a tool with a specific design [10]. In this case, we can find studies where a force control is not used, but only a single position control is used. However, it is necessary to know the whole geometry of the part and to have a computer-aided design and manufacturing software to generate the right trajectory. It is also necessary to use a tool that absorbs vibrations due to the contact force [11]. Another example can be found in [12], where researchers develop a specific end effector for grinding applications. In this study, an abrasive belt and a force-controlled grinding tool mounted in the end effector are used to improve finishing in welding seams. The application of a specific end effector is useful when the workpiece is large. However, a specific device reduces the stiffness and increases the weight in the end effector.

Otherwise, force control can also be carried out actively through feedback with the measured force. This method requires a modification of the control algorithm gains to adapt them to the environmental conditions [13,14,15]. In the case of collaborative robots, some studies have developed sanding applications, but they are based on controlling the torques of the motors [16], which is not usually possible in commercial robots. Other studies, such as [17], combine a force sensor with a laser position sensor. The laser is responsible for keeping the tool in the normal direction to the workpiece, independently of the changing geometric shape. However, this task can also be performed by the force controller. End effector torques or tilt angle can be controlled through machine learning, allowing the user to teach the desired route with significant precision [18].

In [19], the authors presented a complete analysis of the use of an inner/outer loop force control in collaborative robots, from which it is extracted that the best results will be obtained for an inner velocity loop and an outer force loop with a Proportional-Integral with Velocity feedback algorithm (PIV), or a Proportional with Feedforward algorithm (P + FF). Under the conditions of this study, one of the main contributions of this work is that the force control does not depend on the environment. This allows obtaining of the reference value without the need to change the gains of the control algorithm when the task is performed on different materials. These are the algorithms that are going to be used and tested in the present work.

In addition to force control, productivity is an important feature to be improved with the automation of these processes. The most basic way to measure the material removal rate (MRR) is through the Preston equation (Equation (1)).

(1) MRR=k·P·V,

where P is the contact pressure, V is the feed rate of the tool, and k is the Preston coefficient, which is determined experimentally and depends on the material, abrasive, and lubrication, among other factors [20]. However, other works provide more complex mathematical models that allow obtaining of the minimum number of passes and the characteristics of the abrasive material that should be used to obtain the desired roughness [11,21]. An experimental investigation was developed in [22], from which the parameters that affect the surface quality in an industrial robot (no collaborative) polishing could be obtained. From this work, it was concluded that the geometry of the workpiece and the cutting speed do not contribute significantly to the roughness response. However, an increase in the feed rate will generate an increase in the surface roughness value.

Another important aspect in Smart Manufacturing Systems is the determination and optimization of the process parameters to eliminate wastage of resources, especially materials and energy. In [23], the authors used teaching–learning-based optimization and bacterial foraging optimization methods. They obtained the optimum values of cutting speed, feed rate, and depth-of-cut to achieve the lowest surface roughness parameters and cutting temperature.

In a similar way, the authors in [24] used a factorial procedure to characterize the experimental robotic system, predicting the attainable manufacturing tolerances, and allowing the study of the main constraints in the machining of relatively soft materials.

Despite the advances in productivity and optimization of parameters in sanding tasks, it is necessary to study the capacity of collaborative robots in these applications. For this reason, this article is dedicated not only to demonstrating that it is possible to perform operations that imply additional efforts with cobots, but that these operations can be improved with a study that includes the control loops used and the main characteristics of the process and the environment.

One of the situations that this study has revealed is what we have colloquially called “the process saturation concept”. This concept is directly related to the conditions of execution of the operation. In this case, it is an orbital sanding process, where effort is being applied between the sandpaper tool and the part, while movement is carried out on the work surface (cut feed) at the same time as the sandpaper rotates around its axis (cutting movement). Once these three parameters (force, cutting speed, and feed rate) have been set, the grain size is the decisive element that ultimately determines the surface quality achieved. In other words, once the surface has been completely sanded, improving the finish using the same grain size would hardly imply any improvement (beyond the almost negligible effect that the wear of the grains themselves could have). This means that, once a cutting speed is set (orbital sanding usually employs motors without speed variation), it is possible to:

- Adjust the applied force, as long as it is sufficient for the grains to remove the material, looking for the best selection for the combined, control algorithm—characteristics of the robot (e.g., taking into account the sampling frequency);
- Increase the feed rate to improve productivity.

The minimum force required together with the maximum cut feed rate would be the “optimal” values. Any variation on them that does not prevent reaching the quality provided by the selected grain size would mean “saturating the process”. That is, reprocessing the same area without any improvement.

This article is structured as follows. Section 2 describes the materials, experimental bench, and the design of experiments used. Section 3 shows the results and discussion of the experiments developed with the collaborative robot. Finally, Section 4 presents conclusions and future work.

## 2. Materials and Methods

### 2.1. Experimental Setup

**Figure 1:**
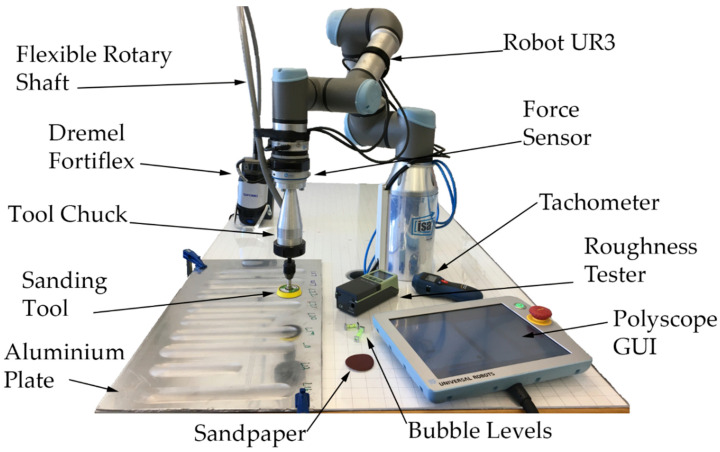
Experimental setup.

The sanding operation to be performed consists of a straight movement on the XY plane, travelling 189 mm along the +Y direction of the robot. The experimental bench can be seen in Figure 1. A collaborative robot from the company “Universal Robots”, UR3 (Universal Robots A/S, Odense, Denmark), is used, with a maximum load of 30 N. A force sensor “OnRobot HEX-EB165” (OnRobot A/S, Odense, Denmark) with 6 degrees of freedom is docked at the end effector of the robot. The data received from the sensor measurements have an accuracy of 0.001 N and a signal noise of 0.2 N in Z, according to its data sheet. The measurement of variables such as positions, speeds, forces, and torques is carried out through the robot controller. These are sent in real-time to the computer via ethernet with a sampling period of 8 ms. Data acquisition is made through the “Labview” software (version 2017, National Instruments, Austin, TX, USA), to be later processed using the “MATLAB” software (version 2019, MathWorks Inc., Natick, MA, USA).

The sanding tool consists of a commercial 50 mm diameter disc with an adherent surface at its bottom, which allows the exchange of sandpaper for each experiment. The sanding tool is driven by a “Dremel” with a flexible shaft. To expand the range of tool diameters that can be clamped and cutting power, the flexible shaft was replaced by one from the German company Wolfcraft (Wolfcraft GmbH, Kempenich, Germany). Wolfcraft limits the revolutions for their flexible shafts to 3500 rpm. However, to couple the new shaft to the Dremel and to mount an industrial tool clamping system on the UR3, with some common parts with a BT30-ER11 tool holder (Ferretería UNCETA S.A, Elgoibar, Spain), new self-made parts were necessary.

The final system planned forced a reduction in revolutions to values below 1500 rpm. The self-made tool chuck that holds and allows the rotation of the sanding tool can be seen in Figure 1. This tool chuck is screwed to the interchangeable base of the robot tip.

### 2.2. Design of Experiments

The input variables of the design of experiments planned for this study are shown in Table 1. Two possibilities are allowed for the control algorithm—PIV and P + FF. The magnitudes for the reference force used are 2.5 and 5 N. The materials on which the sanding operation is executed are steel, brass, aluminium, wood, and PVC (polyvinyl chloride)—materials with very different properties. These are commercial materials, and they are supplied pre-treated. This means that the initial roughness in some of them (see Table 1) is better than what can be achieved with a P600 grain size. This research aims to prove that collaborative robots can be used in operations that imply additional efforts, maintaining a constant $R_{a}$ value under the selected working conditions. The final industrial function for the processed surface, and whether the roughness for that purpose should be greater or less than the original one, remains outside of this study.

In order to reduce the number of experiments, the cutting speed was set to 1070 rpm (below 1500 rpm), and feed rate was set to 5 mm/s (300 mm/min); this last value was decided consistently with the cutting speed and with the sampling period. The grain size selected for the sandpaper was P600. It should be noted that the diameter of the tool, the grain size, and the magnitude of the reference force significantly affect the set of forces required in the process, which is why their values are in concordance with the limitations of the collaborative robot used.

The algorithms and their control gains have been previously determined in the research referred in [19]. These algorithms allow obtaining of the best results in the UR3 robot when an inner velocity loop is used. During the trajectory, the force is controlled in the Z direction, while the movement is controlled in the X and Y direction employing the velocity loop. The feed rate (300 mm/min) is active in the Y direction and is zero velocity in the X-direction. Due to the type of tests, one single path on a flat surface, and the results of preliminary tests, the velocity loop in the UR3 keeps the variations in feed rate very low. Once completed, all the tests shown in Section 3 “Results and discussions” were calculated for the mean feed rate, which was around 304 mm/min, less than 1.3% variation. These variations are not considered in this study.

For the selected variables, it is necessary to perform 20 experiments. Two repetitions were made with a total of 40 experiments. In each experiment, a new path is made in the corresponding material, using a new sanding disc each time. Before executing the sanding operations and with the sanding tool stopped, a couple of bubble levels are used, one in the direction of the *X*-axis and the other in the direction of the *Y*-axis, to leave the sanding disc parallel to the work surface in each test. Due to the type of machining performed in the tests, a considerable inclination of the sanding disc could cause decompensation on the resultant cutting forces between the area that works in accordance and the area that works in opposition. This would accentuate the different surface finish between both areas, the wear of the tool [25], and most importantly, it would cause an imbalance in the tool, increasing oscillations and even causing the UR3 to overstress devices of security. However, in our case, the support for the sandpaper is not rigid; therefore, small parallelism deviations between the sandpaper and the surface are easily absorbed by this support.

If the robot is used in production, the arrangement of the tool axis normal to the work surface can be automated by software. In this case, it is necessary to incorporate an orientation correction in the control loop, either by a measurement in real-time or by planning the trajectories to be executed.

Because the cutting speed is set without load, this must be measured in each test. Through a digital tachometer “PCE-DY-65” (PCE Ibérica S.L., Albacete, Spain), it is possible to check the speed differences between the theoretical and the real value once the tool contacts with each different material.

After the execution of the tests the $R_{a}$, arithmetic mean roughness (ISO 4287), is measured at three different points in the machined area: close to the beginning, middle, and the end of the path. The tests only have one travel, the overlap between cutting paths, needed to guarantee the same roughness over the entire surface, which was not included in this study. Based on this criterion, the roughness measurements have been made in the central area to avoid the effect that takes place on the sides of the trajectory due to the variation of the contact force. This effect is caused by the slightly flexible disc that holds the sandpapers. The value of $R_{a}$ finally shown corresponds to the average of the three measurements taken. In the measurements, the “Mitutoyo SJ-201” roughness tester (Mitutoyo Europe GmbH, Neuss, Germany) is used. From the data acquired through the force sensor mounted on the robot’s wrist, the variables calculated for each test are:

- The mean of the force measurements on the *Z*-axis, ${\bar{F}}_{z}$.
- The standard deviation of the measured force, $S_{z}$.
- The maximum percentage deviation, $\Delta max_{z}$, from the reference.
- The minimum percentage deviation, $\Delta min_{z}$, from the reference.
- The number of upper peaks, $N_{upp}$ (>3.5/6 N), represents the number of deviations that exceed the value of the reference force by +1 Newton.
- The number of lower peaks, $N_{low}$ (<1.5/4 N), represents the number of deviations that exceed the value of the reference force by −1 N.
- The force error, $e_{f}$, between the reference value and the mean ${\bar{F}}_{z}$. This relative error is obtained by subtracting the mean of the force measurements to the reference force value divided by the reference force value.

The limit of 1 N to measure the upper and lower peaks was decided after performing several prior experiments with the OnRobot HEX-EB165 force sensor and the UR3. The experiments covered different tasks (machining on soft materials, polishing, and sanding) and all the measures indicated that 1 N was a perfectly demandable value for the system. Like the roughness measurements, in each experiment, the force measurements are divided into three equal intervals, so that for each dependent variable, there is a total of six samples.

### 2.3. Analysis of Variance

Finally, a three-way ANalysis Of VAriance (ANOVA) is performed to evaluate the effect of the variables on roughness and the results of the force control. The variables measured by the force sensor were taken as dependent variables, and as fixed factors, the parameters, type of control, reference force, and material were used. The Shapiro–Wilk test was used to determine if data were normally distributed before analysis. Furthermore, the Levene test was used to assess the homogeneity of variance in each factor group. Both assumptions are corroborated for each combination of groups of the independent variables. Once it has been determined that there are general differences between the means, post hoc tests are performed to determine which variables in each group differ from each other, that is, the tests allow a pairwise comparison. Tukey’s test was used in the paper. The results of the ANOVA are represented by a 95% confidence level (*p* < 0.05).

## 3. Results and Discussions

### 3.1. Effect of the Parameters

The results of the experiments can be seen in Table 2. These values are the means of six samples, three for each repetition. The values between parentheses indicate the standard deviation of the six measurements. Most of them present a good outcome regarding compliance with the reference force. In general, many peaks due to overshoots are also observed in the results. However, the number of lower peaks is greater than the number of upper peaks; this is related to the measured values of the force since their mean values are less than the value of the reference force. It is important to highlight that the measurements shown correspond to the data obtained during the entire time that the tool is in contact with the workpiece, allowing a total amount of approximately 9000 data read.

The results of the multivariate analysis are shown in Table 3 and Table 4. The F-ratio is a test used to evaluate the explanatory power of a group of independent variables on the variation of the dependent variable. If that ratio is large enough, it can be concluded that not all means are equal. To be concise, only groups of variables that had a significant *p*-value (<0.05) are shown. The complete ANOVA results can be found in Table S1.

It is observed that the “Control type” factor does not produce significant differences in the variables of the study. The “Material” factor produces significant differences in the variables, arithmetic mean roughness ($R_{a}$), the standard deviation of the force ($S_{z}$), the maximum percentage deviation ($\Delta max_{z}$), the minimum percentage deviation ($\Delta min_{z}$), the number of upper peaks ($N_{upp}$), and the number of lower peaks ($N_{low}$). The “Reference force” factor produces significant differences in the variables, arithmetic mean roughness ($R_{a}$), mean of the contact force (${\bar{F}}_{z}$), the standard deviation of the force ($S_{z}$), minimum percentage deviation ($\Delta min_{z}$), the number of upper peaks ($N_{upp}$), and the number of lower peaks ($N_{low}$). Furthermore, it should be noted that there is no significant effect due to the interaction between control type with reference force and control type with material. The interaction between reference force and material produces significant differences in the variables, arithmetic mean roughness ($R_{a}$), the standard deviation of the force ($S_{z}$), minimum percentage deviation ($\Delta min_{z}$), the number of upper peaks ($N_{upp}$), and the number of lower peaks ($N_{low}$). Finally, triple interactions between factors only produce significant differences in the arithmetic mean roughness ($R_{a}$).

**Figure 2:**
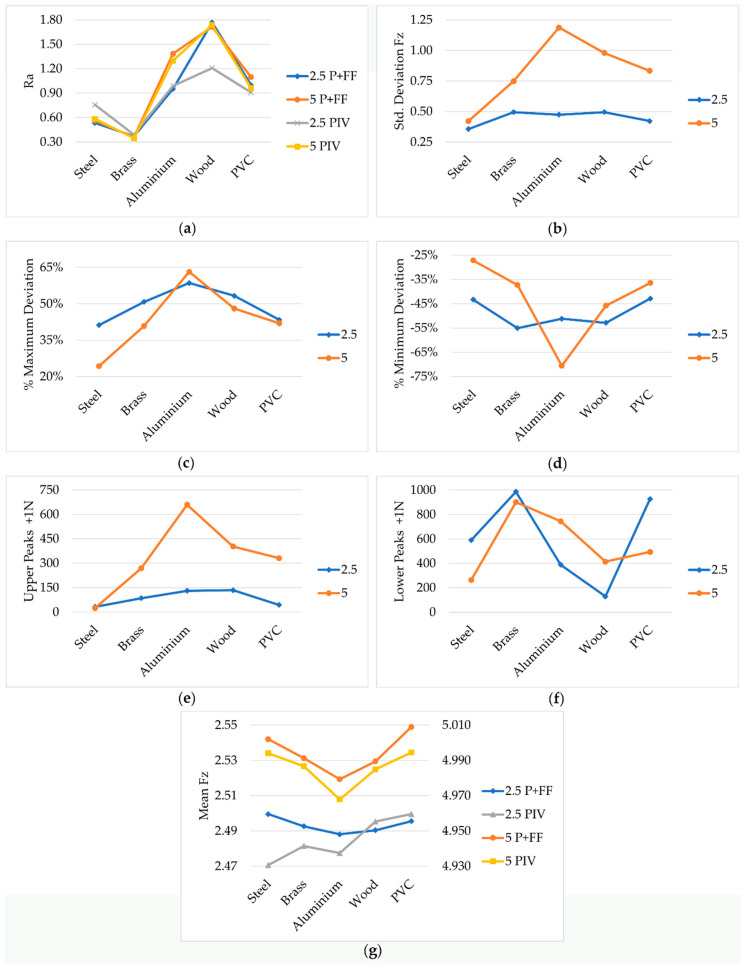
Marginal means. (**a**) Surface roughness, (**b**) standard deviation, (**c**) maximum deviation, (**d**) minimum deviation, (**e**) number of upper peaks, (**f**) number of lower peaks, (**g**) mean of contact force.

In Figure 2, the marginal means of the analysed variables can be observed. Plots show how these variables vary according to the type of material, control type, and reference force.

The marginal means have been calculated in order to visualise, in a better way, the factors that influence the variable plotted in ordinates. This implies that in the case of Figure 2a–d, the means obtained represent four data, two for the P + FF controller and two for the PIV controller. This can be made because the variables shown in ordinates are not affected by the control type.

**Figure 3:**
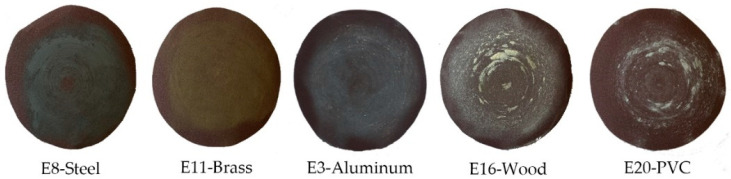
The surface appearance of sanding discs.

In the plot of Figure 2a, something already known becomes evident, such as the direct influence of the type of material on the roughness achieved, when the working conditions remain constant. However, it is interesting to note how in softer materials, according to Young’s modulus (aluminium, wood, and PVC), the application of a greater force on the sandpaper does not significantly improve the finish. This is mainly due to the rapid dulling of the sandpaper, as can be seen in Figure 3, where the surface appearance of the sandpaper discs, selected as examples for each material, can be compared.

Continuing with the observation of Figure 2a, this shows how all materials maintain a trend and very similar values, in the two reference forces, which reinforces the hypothesis that the operation is in a “state of saturation”. With the chosen cutting conditions and grain size, an increase in force does not cause significant improvements. In this variable, $R_{a}$, Tukey’s test for post hoc analysis (pair comparison) indicated significative differences between surface roughness obtained in all materials except for steel with brass (*p* = 0.137) and PVC with aluminium (*p* = 0.122).

Another interesting aspect is the higher standard deviation in the softer materials (aluminium, wood, PVC; Figure 2b) when a force of 5 N is applied. The dulling effect, mentioned above, contributes to increasing friction, which causes a greater separation concerning the reference force that must be continuously compensated. In this variable, Tukey’s test for post hoc analysis (pair comparison) indicated significative differences between standard deviation obtained in steel with aluminium (*p* = 0.000) and steel with wood (*p* = 0.001).

In Figure 2c,d, we can observe the maximum and minimum deviations regarding the reference force. In general, it is detected that the worst behaviour is when a force of 2.5 N is applied, regardless of the control algorithm used—P + FF or PIV. It is important to highlight the high deviations obtained in aluminium. This behaviour has a direct relationship with the working conditions, cutting speed, and feed rate. When the right conditions required by the material deviate further from those used in the tests, the greater the minimum deviations are. In this variable, the post hoc test indicates significative differences between the minimum deviations obtained in steel and aluminium (*p* = 0.041). In these variables, Tukey’s test for post hoc analysis (pair comparison) showed significative differences between the maximum deviations obtained in steel with aluminium (*p* = 0.000), steel with wood (*p* = 0.005), aluminium with brass (*p* = 0.027), and aluminium with PVC (*p* = 0.004). Significative differences between minimum deviations were obtained in steel with aluminium (*p* = 0.000), steel with wood (*p* = 0.018), aluminium with brass (*p* = 0.012), and aluminium with PVC (*p* = 0.000).

In Figure 2e,f, the number of upper peaks is bigger in soft materials when a force of 5 N is used. In contrast, the number of lower peaks is bigger for a force of 2.5 N in the most rigid materials. This is directly related to the stiffnesses of these materials. If the material is soft, it affects the dynamics of the process, so that a greater number of oscillations will be obtained at a lower frequency. On the other hand, in hard materials, there will be a lower number of oscillations, but at a higher frequency. In these variables, Tukey’s test for post hoc analysis revealed significative differences between the number of upper peaks obtained in steel with aluminium (*p* = 0.001) and steel with wood (*p* = 0.001). Significative differences between the number of lower peaks were found in steel with aluminium (*p* = 0.001) and steel with wood (*p* = 0.003).

Finally, in Figure 2g, the fact that the material does not affect the mean force (${\bar{F}}_{z}$) corroborates the results obtained in work [19]. In that work, it was deduced that when using an inner velocity loop, the value of the force in the steady state does not depend on the material stiffness. However, it does affect the dynamics of the process. On the other hand, the reference force and the type of control influence the mean of the contact force. In general, if the P + FF control is used, the mean is closer to the reference value. This effect is more evident with a reference force of 5 N.

### 3.2. Graphs of Force Response and Surface Aspects

For this section, the most significant response graphs have been selected. The full results can be found in the Supplementary Materials, Figures S1–S15. It is important to mention that the empty entry times are different in each test since they are influenced by the thickness of the material and by the speed of the first impact, which is a function of the reference force. Additionally, the noise at the input is a product of the no-load noise (0.2 N) of the sensor plus the vibrations produced by the revolution of the tool. The noise due to the vibrations is important because it affects all the measures; it behaves like a systematic error. To decrease these vibrations, the Dremel and/or the method used to transmit the torque (flexible shaft) should be changed.

**Figure 4:**
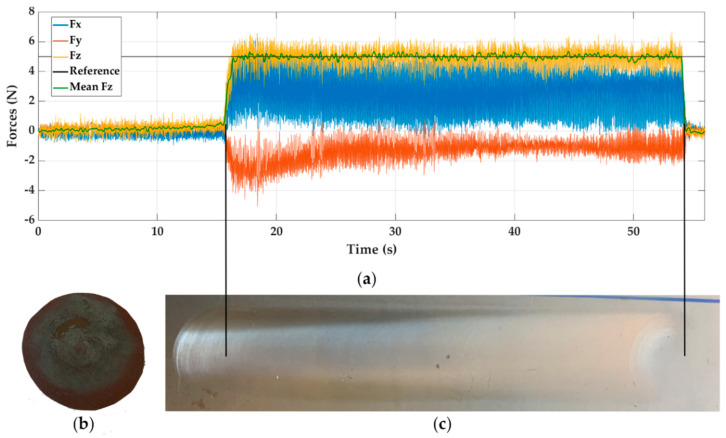
Experiment E6, sanding steel with P + FF control and reference force of 5 N. (**a**) Force response, (**b**) sandpaper aspect, and (**c**) visual surface finish.

In Figure 4a, the test that showed the best behaviour of the force *Fz* is shown (yellow colour). This was obtained working on steel, using a P + FF control, and with a reference force of 5 N (black line). A green curve within the force/time graph of the mean force *Fz* is shown to visualise the control method effects better. Figure 4b shows the appearance of the sandpaper used after processing the test. In Figure 4c, the trajectory followed by the sanding tool has been included, in parallel with the measured forces. This way, it is easy to relate the marks left by the tool on the material with the variation obtained in the forces. The vertical lines in black represent the start and endpoint of the toolpath.

It is interesting to notice how the transition at the end of the control algorithm (with a duration of less than one second) between the force control and velocity control (once contact is finished) makes the marks left less intense, even incomplete. To solve this, it would be necessary to keep the tool in this area longer when the trajectory has finished.

Querying the specific numerical values for this test in Table 2, we can see that this experiment is one of the most stable, since it presents the minimum upper and lower peaks in addition to achieving one of the smallest force errors. This corroborates the effect of material stiffness on the process dynamics. The minimum values of the oscillations are related to the noise of the force sensor.

For the case of the forces *Fx* and *Fy*, a greater number of oscillations are observed, being greater for the direction in the *X*-axis. The value of the force in the *Y*-axis is due to the process friction. However, in the case of the *X*-axis, this happens due to the tangential forces acting on the sanding process. According to the spatial configuration used in the robot, the force *Fx* is only supported by one of the robot joints with less capacity. Therefore, as it is a more flexible joint, it generates a great number of oscillations.

**Figure 5:**
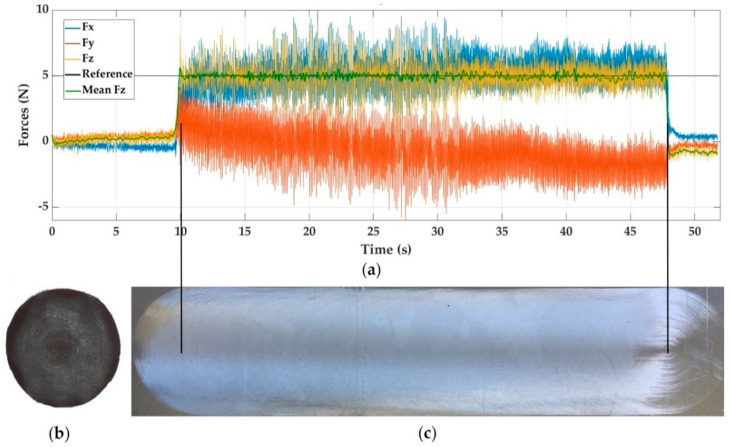
Experiment E2, sanding aluminium with P+FF control and reference force of 5 N. (**a**) Force response, (**b**) sandpaper aspect, and (**c**) visual surface finish.

Figure 5a shows the force response graph for E2. In Figure 5c, you can see the surface result for the aluminium sanding test using a P + FF controller and a reference force of 5 N. Figure 5b evidences the state of the sandpaper after the operation.

In general, the force response between steel and aluminium are very similar; however, in aluminium, more oscillations are observed in the final part of the trajectory. This is supported with the previous results of the ANOVA analysis in which aluminium had the highest maximum and minimum percentage deviation from the reference value. It can also be seen that, due to the lower stiffness of the material, the dynamics are different, so greater amplitude oscillations appear, but with a lower frequency (closer peaks).

Furthermore, the forces *Fx* and *Fy* are of greater magnitude than in the case of sanding steel, being responsible for the dulling of aluminium that induces a greater friction force. This was also ratified by the amount of powdered chips left on the treated surface.

**Figure 6:**
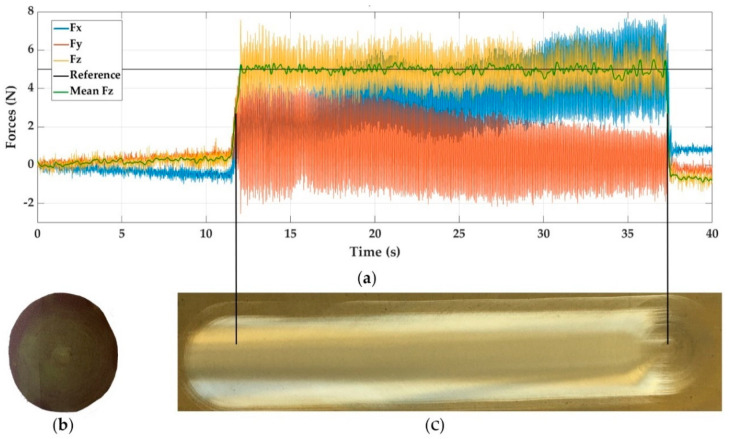
Experiment E12, sanding brass with PIV control and reference force of 5 N. (**a**) Force response, (**b**) sandpaper aspect, and (**c**) visual surface finish.

In the case of sanding brass, Figure 6a shows the response graph and Figure 6c the surface appearance obtained after using a PIV controller with a reference force of 5 N.

In E12, it can be noticed that force *Fz* presents an average value of oscillations between the steel and aluminium, which is in accordance with the material rigidity. Furthermore, it can be observed how in brass, the overshoots are of less intensity than those in aluminium.

Regarding the appearance of the sandpaper, it contains a greater amount of residual material. This is because sanding on brass produces a residue, powder type, that dyes sandpaper.

**Figure 7:**
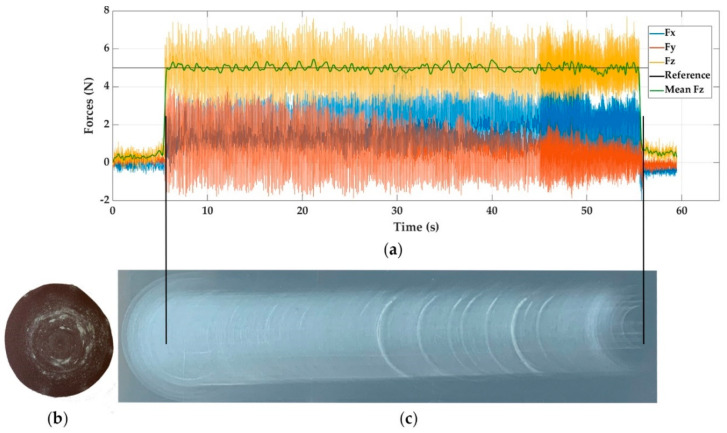
Experiment E20, sanding PVC with PIV control and reference force of 5 N. (**a**) Force response, (**b**) sandpaper aspect, and (**c**) visual surface finish.

**Figure 8:**
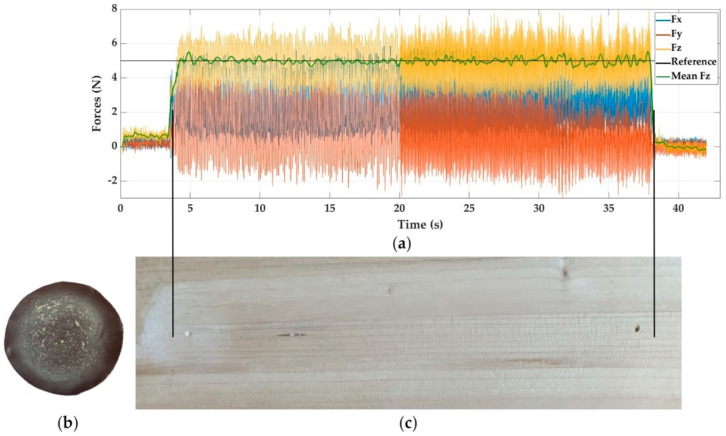
Experiment E14, sanding wood with P+FF control and reference force of 5 N. (**a**) Force response, (**b**) sandpaper aspect, and (**c**) visual surface finish.

Continuing with the softer materials, in Figure 7 can be seen the response graph (a), the aspect of the PVC surface after sanding it with a PIV controller and 5 N as a reference force (c), and the final condition of the sandpaper (b). On the other hand, Figure 8a shows the response graph for sanding wood with a P + FF controller and 5 N as a reference force too. Figure 8b,c show the wear produced in the sandpaper and the surface appearance left, respectively.

The response graphs of PVC and wood are very similar; they present great stability in the force in the *Z*-axis, and the oscillations keep constant around the reference force, but of greater amplitude than in the case of steel. It can be confirmed that regardless of the type of control (P + FF or PIV), the behaviour is similar. The remarkable thing, according to the selected cutting conditions for the PVC, is the dulling that the sandpaper suffers, which generates marks on the sanded surface until the stuck material comes loose. This is due to softening of the material by heat. It should be noted that both examples are for a reference force of 5 N. In the case of reference forces of 2.5 N, dulling exists, but it is lower.

### 3.3. Effect of Feed Rate

Since the reference force does not have a significant effect on the surface roughness, it is interesting to test what effects the feed speed will produce. This way, the sanding process can be optimised without overloading the contact forces with the robot. Table 5 shows the experiments carried out on brass with a PIV control and a reference force of 2.5 N. In these tests, the cut feed is varied in four levels—300 (repeating E11), 450, 600, and 900 mm/min.

As explained in the Introduction under the name of the “process saturation concept”, it can be noticed how a change in the cut feed does not generate a significant change in the surface roughness on the brass. Additionally, it can be verified that the results of the force measurements in E21 are similar to the previous experiment, E11.

**Figure 9:**
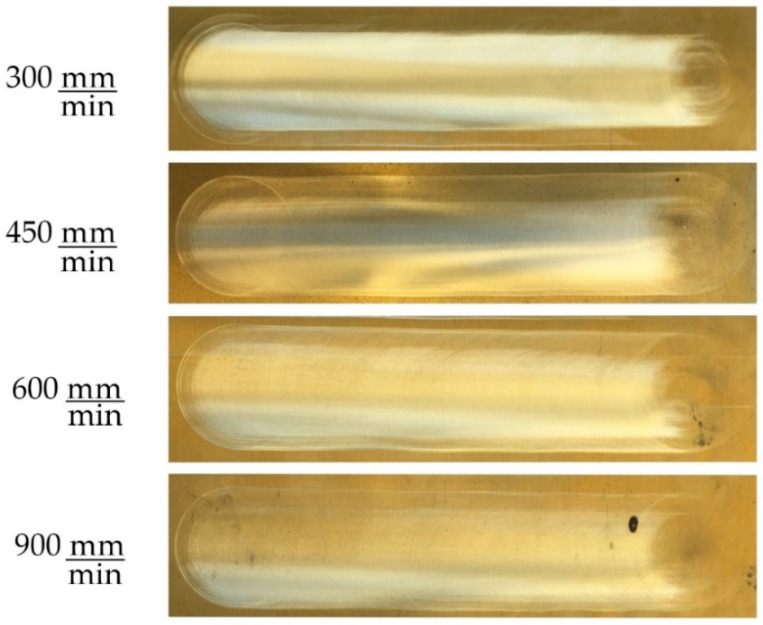
Results for cut feed variation on brass.

In Figure 9, the surface appearance of the experiments with speed variation is shown. It should be noted that the pictures have been taken separately, with a different position and orientation of the camera; hence, the changes in brightness are appreciated. However, what is really important are the values obtained, shown in the table above.

Given the value of $R_{a}$ measured, practically the same for the E21, E22, E23, and E24 tests, this allows us to select a higher feed rate for the same reference force, type of control, cutting speed, and tool diameter. This way, it is possible to increase the productivity of the process.

Comparing the most extreme values of the tests in Figure 9, the cut feed in E24 is tripled with respect to E21, achieving the same value of $R_{a}$, 0.44 µm. The rest of the parameters hardly change, according to what they represent. The standard deviation of the measured force, $S_{z}$, remains at similar levels, as do the maximum and minimum percentage changes, $\Delta max_{z}$, $\Delta min_{z}$. The most significant difference is found in the number of upper peaks $N_{upp}$ (>3.5/6 N) and lower peaks $N_{low}$ (<1.5/4 N), where a considerable improvement is observed in the case of E24, with higher cutting speed. As can be noticed in the different tests shown, the noise in the force sensor and the intrinsic characteristics of the process itself (where forces are mixed with cycloid movements) generate many oscillations, as well as lower and upper peaks. However, the improvement in E24 is considerable, due to the inertia of the robot during the cut feed and the influence of dynamic friction speed, among other things.

Following the comparison between E21 and E24, if we apply the Preston formula (Equation (1)) maintaining the same pressure in the two tests (P), as well as the same material and same abrasive (K), the material removal rate (MMR) in the E24 assay triples the rate achieved in E21.

### 3.4. Comparison with a Standard Industrial Robot

Industrial robots are widely used in grinding and sanding processes with excellent performance. In order to have reference values, it is interesting to contrast the results of the collaborative robot UR3 with standard industrial ones, working with similar process conditions.

Chen et al. [26] used a Kuka KR60-3 to perform several experiments on carbon fibre composite material with different cutting conditions. Among these conditions, they used a rotational speed of 3000 rpm, cut feed of 30 mm/s (1800 mm/min), Z force of 10 N, sandpaper grain of P600, and an inclination of the tool axis of 10°, obtaining a final $R_{a}$ of 1.77 µm.

Chen et al. sand the outer layer of the composite material. The type of resin is not named in the article, but we can make a reasonable comparison between plastic materials. The UR3 has been used to sand PVC with the same Z force, same grain size, and adapting the cutting speed to 1000 rpm and the consequent feed rate to 600 mm/min to keep the same proportion as them. However, taking into account the low rigidity of the UR3 and the increase in Z force and feed rate, the tool axis tilt angle used to avoid instability is 7°. The control algorithm used is P + FF.

The value of the $R_{a}$ parameter achieved is 0.45 µm, being $e_{f}$ = 0.07%, ${\bar{F}}_{z}$ = 10.007, $N_{upp}$ = 243, and $N_{low}$ = 320. Certainly, the main reason for the differences between the $R_{a}$ values are the characteristics of the plastics, but the results of the experiment confirm the feasibility of the operation with a cobot.

**Figure 10:**
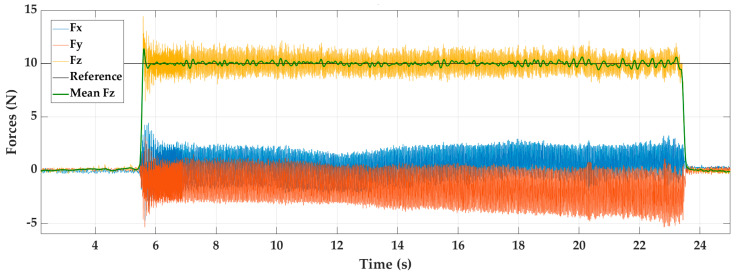
Force response for UR3 sanding wood with a sandpaper grain size of 400.

Nagata et al. [13], with a Kawasaki FS20N robot for sanding wood (oak), employed in their last pass a Z force of 10 N, a feed rate of 30 mm/s (1800 mm/min), and a grain size of 400. The UR3 has been used with a PIV control algorithm, the same Z force, the same size of grain, and a feed rate adapted to 600 mm/min. In these conditions, the $R_{a}$ achieved was 1.64 µm, with an $e_{f}$ = 0.13%, ${\bar{F}}_{z}$ = 10.013, $N_{upp}$ = 489, and $N_{low}$ = 687. Once again, the type of wood is not the same and Nagata et al. apply three passes with 80, 220, and finally, 400 grain size to obtain the final result of 1 µm. However, the process can be made with the collaborative robot, and the force response is acceptable, as can be seen in Figure 10.

Nevertheless, the main differences between the cobots and the standard industrial robots are the much smaller torque and the low rigidity that collaborative robots have. These differences limit the achievable performances, decreasing productivity significantly. A clear example of the repercussions of these differences is the weight and dimensions of the tool (sandpaper disc diameter, for example) that the cobot can bear.

## 4. Conclusions

The capability to perform a sanding process with a collaborative robot has been demonstrated through various experiments on different materials. What has also been confirmed is the importance of knowing what the best type of control in the combination is: inner control loop for robot movements and outer loop to control force in the robot. It should be noted that the force control with an inner velocity loop allows obtaining of good results in contact force control tracking, since, as it has been seen, this type of control in the UR3 robot is not affected by the rigidity of the processed material [19].

On the other hand, there has been an opportunity to test how the stiffness of the material (hardness) only influences the dynamics of the process, generating a greater or lesser number of peaks with respect the reference force, as indicated by the ANOVA results.

From the analysis of variance, it follows that the type of force control algorithm—PIV or P + FF—does not have significant effects on the performance of the sanding process using a collaborative robot like the UR3. The effective difference would be found with more complex cutting paths. In these cases, both algorithms will be valid, but work conditions (especially feed rate) should be fitted to achieve the desired roughness. These adjustments will have a direct impact on the productivity reached.

Furthermore, increasing the force does not have a significant effect on the surface finish either. That means the surface finish is determined by the type of material and the grain size of the sandpaper only, as long as the force is enough for the right application of sanding. However, a triple interaction between the factors—control type, reference force, and material—produces a significative difference in the arithmetic mean roughness. The worst values are obtained in soft materials with a P+FF control and a reference force of 5 N.

Due to the low effect (almost null) of the reference force value, it was decided to perform an analysis to check the effect of the feed rate according to what was stated in the Introduction under the name of the “process saturation concept”. This analysis allowed us to corroborate that the process was in a “state of saturation”, with which a productivity improvement could be sought simply by adjusting the cutting conditions. As a demonstration, it was decided to increase one of the cutting conditions, the cut feed. The results maintain the same value for the surface finish of brass, 0.44 µm, tripling productivity, and keeping the force level at its minimum value. Unlike the study in [22], the level of roughness obtained with the sanding process is not affected by an increase in the feed rate, in a “saturated state”. This is the main outcome from this work because it will allow optimizing of the process parameters in future sanding tasks with a collaborative robot.

A comparison between industrial and collaborative robots showed that the latter can perform the same sanding operation with similar results of surface roughness. Therefore, these experiments confirm the feasibility of these operations with a cobot. However, the main limitation will be the payload capacity of the cobots.

As future works, it would be interesting to study in depth what parameters can be modified in the force control and what types of impact control algorithms can be used to minimise the effect caused by the sanding tool on the entry and exit of the trajectory. In addition, the controller might require additional variables such as the vibration effects introduced by the dynamic components. Another important aspect of developing would be to analyse the stability of the sanding process when it also has the option to vary the cutting speed. Finally, it is important to include optimization methods to determine the process parameters that allow for obtaining the best roughness surface results.

## Figures and Tables

**Table 1:** Design of the experiments.

Control Algorithm	Reference Force	Materials	$\mathrm{Initial}\;R_{a}$ (μm)	Feed Rate	Cut Feed	Sandpaper Grain
PIV	2.5 N	Steel Brass Aluminium Wood PVC	1.30 0.38 0.18 2.10 0.17	300 mm/min	1070 rpm	P600
P + FF	5 N					

**Table 2:** Experimental results.

N° Exp	Control	Ref. Force (N)	Material	$R_{a}$ (μm)	Mean ${\bar{F}}_{z}$ (N)	Std. Dev. $S_{z}\;(N)$	$\Delta max_{z}$ (%)	$\Delta min_{z}$ (%)	$N_{upp}$ >3.5/6 (N)	$N_{low}$ <1.5/4 (N)	$e_{f}$ (%)
E1	P + FF	2.5	Aluminium	0.95 ^a^	2.488 ^a1^	0.495 ^a^	60 ^a11^	−53 ^a11^	209 ^a1^	186 ^a1^	0.48 ^a^
				(0.34)	(0.0021)	(0.230)	(25)	(12)	(129)	(111)	(0.008)
E2	P + FF	5	Aluminium	1.38 ^a^	4.979 ^a1^	1.000 ^a^	59 ^a11^	−61 ^a11^	836 ^a1^	973 ^a1^	0.41 ^a^
				(0.36)	(0.027)	(0.283)	(16)	(17)	(222)	(213)	(0.005)
E3	PIV	2.5	Aluminium	0.99 ^a^	2.477 ^a1^	0.458 ^a^	57 ^a11^	−50 ^a11^	53 ^a1^	591 ^a1^	0.90 ^a^
				(0.21)	(0.017)	(0.160)	(13)	(13)	(46)	(101)	(0.007)
E4	PIV	5	Aluminium	1.30 ^a^	4.968 ^a1^	1.402 ^a^	71 ^a11^	−67 ^a11^	483 ^a1^	515 ^a1^	0.64 ^a^
				(0.17)	(0.039)	(0.485)	(23)	(23)	(297)	(284)	(0.008)
E5	P + FF	2.5	Steel	0.53 ^b^	2.500 ^a2^	0.389 ^a^	40 ^a2^	−46 ^a2^	45 ^a2^	455 ^a2^	0.02 ^a^
				(0.12)	(0.023)	(0.453)	(20)	(21)	(85)	(74)	(0.009)
E6	P + FF	5	Steel	0.56 ^b^	5.001 ^a2^	0.453 ^a^	23 ^a2^	−29 ^a2^	27 ^a2^	480 ^a2^	−0.04 ^a^
				(0.09)	(0.019)	(0.160)	(6)	(11)	(31)	(188)	(0.004)
E7	PIV	2.5	Steel	0.76 ^b^	2.471 ^a2^	0.324 ^a^	42 ^a2^	−41 ^a2^	21 ^a2^	725 ^a2^	1.18 ^a^
				(0.15)	(0.037)	(0.171)	(19)	(17)	(46)	(153)	(0.015)
E8	PIV	5	Steel	0.58 ^b^	4.994 ^a2^	0.395 ^a^	26 ^a2^	−25 ^a2^	22 ^a2^	48 ^a2^	0.12 ^a^
				(0.20)	(0.018)	(0.079)	(6)	(3)	(22)	(21)	(0.004)
E9	P + FF	2.5	Brass	0.37 ^b^	2.494 ^a^	0.504 ^a^	52 ^a12^	−50 ^a12^	102 ^a^	1037 ^a^	0.29 ^a^
				(0.05)	(0.044)	(0.215)	(35)	(9)	(176)	(174)	(0.018)
E10	P + FF	5	Brass	0.36 ^b^	4.991 ^a^	0.627 ^a^	35 ^a12^	−34 ^a12^	159 ^a^	915 ^a^	0.17 ^a^
				(0.08)	(0.024)	(0.103)	(4)	(2)	(103)	(142)	(0.005)
E11	PIV	2.5	Brass	0.39 ^b^	2.486 ^a^	0.513 ^a^	50 ^a12^	−60 ^a12^	68 ^a^	937 ^a^	0.74 ^a^
				(0.07)	(0.046)	(0.170)	(13)	(6)	(117)	(128)	(0.018)
E12	PIV	5	Brass	0.35 ^b^	4.987 ^a^	0.942 ^a^	47 ^a12^	−40 ^a12^	379 ^a^	888 ^a^	0.27 ^a^
				(0.10)	(0.033)	(0.399)	(20)	(11)	(279)	(271)	(0.007)
E13	P + FF	2.5	Wood	1.77 ^c^	2.490 ^a1^	0.527 ^a^	57 ^a1^	−53 ^a1^	208 ^a^	145 ^a1^	0.39 ^a^
				(0.29)	(0.010)	(0.304)	(28)	(15)	(245)	(162)	(0.004)
E14	P + FF	5	Wood	1.71 ^c^	4.989 ^a1^	0.890 ^a^	43 ^a1^	−42 ^a1^	303 ^a1^	349 ^a1^	0.21 ^a^
				(0.49)	(0.017)	(0.430)	(14)	(10)	(296)	(269)	(0.003)
E15	PIV	2.5	Wood	1.21 ^c^	2.495 ^a1^	0.471 ^a^	49 ^a1^	−53 ^a1^	61 ^a1^	114 ^a1^	0.19 ^a^
				(0.15)	(0.019)	(0.223)	(17)	(20)	(111)	(148)	(0.007)
E16	PIV	5	Wood	1.74 ^c^	4.985 ^a1^	1.073 ^a^	53 ^a1^	−49 ^a1^	502 ^a1^	479 ^a1^	0.30 ^a^
				(0.43)	(0.022)	(0.515)	(18)	(20)	(383)	(413)	(0.004)
E17	P + FF	2.5	PVC	1.00 ^a^	2.496 ^a^	0.432 ^a^	42 ^a12^	−43 ^a12^	32 ^a^	1011 ^a1^	0.18 ^a^
				(0.26)	(0.021)	(0.226)	(16)	(16)	(36)	(117)	(0.009)
E18	P + FF	5	PVC	1.10 ^a^	5.009 ^a^	0.938 ^a^	44 ^a12^	−37 ^a12^	407 ^a^	723 ^a1^	−0.18 ^a^
				(0.25)	(0.029)	(0.558)	(23)	(17)	(428)	(467)	(0.006)
E19	PIV	2.5	PVC	0.91 ^a^	2.500 ^a^	0.415 ^a^	45 ^a12^	−43 ^a12^	57 ^a^	842 ^a1^	0.02 ^a^
				(0.07)	(0.021)	(0.205)	(16)	(16)	(69)	(50)	(0.009)
E20	PIV	5	PVC	0.96 ^a^	4.995 ^a^	0.736 ^a^	40 ^a12^	−36 ^a12^	255 ^a^	265 ^a1^	0.11 ^a^
				(0.18)	(0.023)	(0.386)	(16)	(14)	(284)	(284)	(0.005)

Same superscript letters = no statistically significant difference. a≠b≠c in the same columns indicate significant differences according to Tukey’s test (*p* < 0.05). Same superscript numbers = no significant difference. 1≠2 means a significant difference between them, but not with the others in the same column.

**Table 3:** ANOVA results (part one).

Source of Variation	Variable	Sum of Squares	df	Mean Square	F-Ratio	*p*-Value
Control Type	$R_{a}$	0.094	1	0.094	1.677	0.198
	${\bar{F}}_{z}$	0.002	1	0.002	2.951	0.089
	$S_{z}$	0.054	1	0.054	0.568	0.453
	$\Delta max_{z}$	112.937	1	112.937	0.374	0.542
	$\Delta min_{z}$	234.799	1	234.799	0.970	0.327
	$N_{upp}$	1491.075	1	1491.075	0.034	0.853
	$N_{low}$	69.008	1	69.008	0.002	0.969
	$e_{f}$	1.927	1	1.927	2.498	0.117

**Table 4:** ANOVA results (part two).

Source of Variation	Variable	Sum of Squares	df	Mean Square	F-Ratio	*p*-Value
Reference Force	$R_{a}$	0.411	1	0.411	7.320	0.008
	${\bar{F}}_{z}$	187.630	1	187.630	256,585.617	0.000
	$S_{z}$	4.453	1	4.453	47.012	0.000
	$\Delta max_{z}$	1004.402	1	1004.402	3.325	0.071
	$\Delta min_{z}$	949.822	1	949.822	3.922	0.050
	$N_{upp}$	1,474,305.008	1	1,474,305.008	34.087	0.000
	$N_{low}$	1,620,990.075	1	1,620,990.075	35.571	0.000
	$e_{f}$	1.674	1	1.674	2.169	0.144
Material	$R_{a}$	22.432	4	5.608	99.956	0.000
	${\bar{F}}_{z}$	0.006	4	0.001	1.944	0.109
	$S_{z}$	2.629	4	0.657	6.938	0.000
	$\Delta max_{z}$	10,264.652	4	2566.163	8.496	0.000
	$\Delta min_{z}$	9325.331	4	2331.333	9.627	0.000
	$N_{upp}$	932,522.883	4	233,130.721	5.390	0.001
	$N_{low}$	922,109.617	4	230,527.404	5.059	0.001
	$e_{f}$	4.102	4	1.025	1.329	0.264
Control Type Reference Force	$R_{a}$	0.010	1	0.010	0.180	0.673
	${\bar{F}}_{z}$	2.828·10^−7^	1	2.828·10^−7^	0.000	0.984
	$S_{z}$	0.172	1	0.172	1.814	0.181
	$\Delta max_{z}$	397.822	1	397.822	1.317	0.254
	$\Delta min_{z}$	171.097	1	171.097	0.707	0.403
	$N_{upp}$	90,036.408	1	90,036.408	2.082	0.152
	$N_{low}$	21,253.408	1	21,253.408	0.466	0.496
	$e_{f}$	0.201	1	0.201	0.261	0.611
Control Type Material	$R_{a}$	0.504	4	0.126	2.244	0.070
	${\bar{F}}_{z}$	0.001	4	0.000	0.397	0.810
	$S_{z}$	0.390	4	0.098	1.030	0.396
	$\Delta max_{z}$	98.097	4	24.524	0.081	0.988
	$\Delta min_{z}$	731.837	4	182.959	0.755	0.557
	$N_{upp}$	79,586.883	4	19,896.721	0.460	0.765
	$N_{low}$	138,840.950	4	34,710.238	0.762	0.553
	$e_{f}$	1.807	4	0.452	0.585	0.674
Reference Force Material	$R_{a}$	0.824	4	0.206	3.673	0.008
	${\bar{F}}_{z}$	0.002	4	0.000	0.616	0.652
	$S_{z}$	1.434	4	0.359	3.785	0.007
	$\Delta max_{z}$	1613.502	4	403.375	1.335	0.262
	$\Delta min_{z}$	5328.349	4	1332.087	5.501	0.000
	$N_{upp}$	510,518.617	4	127,629.654	2.951	0.024
	$N_{low}$	495,118.383	4	123,779.596	2.716	0.034
	$e_{f}$	0.995	4	0.249	0.322	0.862
Control Type Reference Force Material	$R_{a}$	0.593	4	0.148	2.642	0.038
	${\bar{F}}_{z}$	0.001	4	0.000	0.469	0.758
	$S_{z}$	0.369	4	0.092	0.975	0.425
	$\Delta max_{z}$	684.011	4	171.003	0.566	0.688
	$\Delta min_{z}$	693.957	4	173.489	0.716	0.583
	$N_{upp}$	250,338.217	4	62,584.554	1.447	0.224
	$N_{low}$	88,352.050	4	22,088.013	0.485	0.747
	$e_{f}$	1.975	4	0.494	0.640	0.635

**Table 5:** Test results for the cut feed variations.

N° Exp	Feed Rate (mm/min)	Ra (μm)	Mean ${\bar{F}}_{z}$ (N)	Std. Deviation $S_{z}\;(N)$	$\Delta max_{z}$ (%)	$\Delta min_{z}$ (%)	$N_{upp}$ >3.5/6 (N)	$N_{Low}$ <1.5/4 (N)	$e_{f}$ (%)
E21	300	0.44	2.4836	0.4302	56	−56	92	5264	0.66
E22	450	0.42	2.4893	0.5834	84	−69	202	2211	0.43
E23	600	0.42	2.4824	0.5646	70	−58	151	7570	0.70
E24	900	0.44	2.4790	0.5140	59	−57	52	2240	0.84

## Data Availability

Data is contained within the article or Supplementary Mterials.

## Supplementary Materials

The following are available online at https://www.mdpi.com/1996-1944/14/1/67/s1, Table S1: ANOVA results, Figure S1: Experiment E1, sanding aluminium with P + FF control and reference force of 2.5 N, Figure S2: Experiment E3, sanding aluminium with PIV control and reference force of 2.5 N, Figure S3: Experiment E4, sanding aluminium with PIV control and reference force of 5 N, Figure S4: Experiment E5, sanding steel with P + FF control and reference force of 2.5 N, Figure S5: Experiment E7, sanding steel with PIV control and reference force of 2.5 N, Figure S6: Experiment E8, sanding steel with PIV control and reference force of 5 N, Figure S7: Experiment E9, sanding brass with P + FF control and reference force of 2.5 N, Figure S8: Experiment E10, sanding brass with P + FF control and reference force of 5 N, Figure S9: Experiment E11, sanding brass with PIV control and reference force of 2.5 N, Figure S10: Experiment E13, sanding wood with P + FF control and reference force of 2.5 N, Figure S11: Experiment E15, sanding wood with PIV control and reference force of 2.5 N, Figure S12: Experiment E16, sanding wood with PIV control and reference force of 5 N, Figure S13: Experiment E17, sanding PVC with P + FF control and reference force of 2.5 N, Figure S14: Experiment E18, sanding PVC with P + FF control and reference force of 5 N, Figure S15: Experiment E19, sanding PVC with PIV control and reference force of 2.5 N.

## References

[1] Kalt E., Monfared R.P., Jackson M.R. Development of an intelligent automated polishing system. Proceedings of the 16th International Conference of the European Society for Precision Engineering and Nanotechnology, EUSPEN 2016.

[2] Walker D.D., Yu G., Bibby M., Dunn C., Li H., Wu H.Y., Zheng X., Zhang P. (2016). Robotic automation in computer controlled polishing. J. Eur. Opt. Soc. Rapid Publ..

[3] Hahnel S., Pini F., Leali F., Dambon O., Bergs T., Bletek T. Reconfigurable Robotic Solution for Effective Finishing of Complex Surfaces. Proceedings of the 2018 IEEE 23rd International Conference on Emerging Technologies and Factory Automation (ETFA).

[4] Tam H.Y., Lui O.C.H., Mok A.C. (1999). Robotic polishing of free-form surfaces using scanning paths. J. Mater. Process. Technol..

[5] Dieste J.A., Fernández-Cuello A., Javierre C., Santolaria J. (2016). Conformal polishing approach: Tool footprint analysis. Adv. Mech. Eng..

[6] Perez-Ubeda R., Gutierrez S.C., Zotovic R., Lluch-Cerezo J. (2019). Study of the application of a collaborative robot for machining tasks. Procedia Manuf..

[7] Qu Y.J., Ming X.G., Liu Z.W., Zhang X.Y., Hou Z.T. (2019). Smart manufacturing systems: State of the art and future trends. Int. J. Adv. Manuf. Technol..

[8] El Zaatari S., Marei M., Li W., Usman Z. (2019). Cobot programming for collaborative industrial tasks: An overview. Rob. Auton. Syst..

[9] Wang K., Dailami F., Matthews J. (2019). Towards collaborative robotic polishing of mould and die sets. Procedia Manuf..

[10] Huang H., Gong Z., Chen X., Zhou L. (2002). Robotic grinding and polishing for turbine-vane overhaul. J. Mater. Process. Technol..

[11] Fernandez A., Jose Antonio D., Javierre C., Jorge S. (2015). Surface Roughness Evolution Model for Finishing Using an Abrasive Tool on a Robot. Int. J. Adv. Robot. Syst..

[12] Li M., Du Z., Ma X., Gao K., Dong W., Di Y., Gao Y. (2020). System design and monitoring method of robot grinding for friction stir weld seam. Appl. Sci..

[13] Nagata F., Kusumoto Y., Fujimoto Y., Watanabe K. (2007). Robotic sanding system for new designed furniture with free-formed surface. Robot. Comput. Integr. Manuf..

[14] Maric B., Mutka A., Orsag M. (2020). Collaborative Human-Robot Framework for Delicate Sanding of Complex Shape Surfaces. IEEE Robot. Autom. Lett..

[15] Liang X., Mohsin I., Xu Y., Yan C., He K. (2020). Robotic Polishing of the Meat Grinder Blade under Path Planning and Controlled Force. IOP Conf. Ser. Mater. Sci. Eng..

[16] Dong Y., Ren T., Hu K., Wu D., Chen K. (2020). Contact force detection and control for robotic polishing based on joint torque sensors. Int. J. Adv. Manuf. Technol..

[17] Wen Y., Hu J., Pagilla P.R. A Novel Robotic System for Finishing of Freeform Surfaces. Proceedings of the 2019 International Conference on Robotics and Automation (ICRA).

[18] Brito T., Queiroz J., Piardi L., Fernandes L.A., Lima J., Leitão P. (2020). A Machine Learning Approach for Collaborative Robot Smart Manufacturing Inspection for Quality Control Systems. Procedia Manuf..

[19] Pérez-Ubeda R., Zotovic-Stanisic R., Gutiérrez S.C. (2020). Force Control Improvement in Collaborative Robots through Theory Analysis and Experimental Endorsement. Appl. Sci..

[20] Guiot A., Pattofatto S., Tournier C., Mathieu L. (2011). Modeling of a Polishing Tool to Simulate Material Removal. Adv. Mater. Res..

[21] Márquez J.J., Pérez J.M., Ríos J., Vizán A. (2005). Process modeling for robotic polishing. J. Mater. Process. Technol..

[22] Padmanabhan S.N., Halil Z., Sun Y., Vu T.T., Yeo S.H., Wee A. (2018). Experimental investigation of Robotic Surface Finishing Using Abrasive Disc. Int. J. Mater. Mech. Manuf..

[23] Mia M., Królczyk G., Maruda R., Wojciechowski S. (2019). Intelligent optimization of hard-turning parameters using evolutionary algorithms for smart manufacturing. Materials.

[24] Iglesias Sánchez I., Ares J.E., González Gaya C., Rosales Prieto V. (2020). A New Approach to the Consideration and Analysis of Critical Factors in Robotic Machining. Appl. Sci..

[25] Zhang S., Zhou K., Ding H., Guo J., Liu Q., Wang W. (2018). Effects of grinding passes and direction on material removal behaviours in the rail grinding process. Materials.

[26] Chen C.-Y., Li J., Zhu Y., Tu L., Weng W. Automatic finishing system research for industrial robot. Proceedings of the 2017 IEEE International Conference on Cybernetics and Intelligent Systems (CIS) and IEEE Conference on Robotics, Automation and Mechatronics (RAM).

