# Supplementary material for: Behavioural Study of the Force Control Loop Used in a Collaborative Robot for Sanding Materials
Source: Materials (Basel). 2020 Dec 25;14(1):67. doi: 10.3390/ma14010067 (PMC7794880; doi:10.3390/ma14010067)
Supplement: Supplementary file 1 [file materials-14-00067-s001.pdf]

materials

MDPI

Behavioural study of the force control loop used in a collaborative robot for sanding materials

Rodrigo Pérez-Ubeda [1,*], Santiago C. Gutiérrez Rubert [1], Ranko Zotovic-Stanisic [2] and Ángel Perles Ivars [3]

[1] Deparment of Mechanical and Materials Engineering, Universitat Politècnica de València, Valencia 46022, Spain; scgutier@mcm.upv.es.
[2] Institute of Industrial Control Systems and Computing, Universitat Politècnica de València, Valencia 46022, Spain; rzotovic@isa.upv.es.
[3] ITACA Institute, Universitat Politècnica de València, Valencia 46022, Spain; aperles@disca.upv.es.
* Correspondence: rodpeub@doctor.upv.es; Tel.: +34 96 387 76 22

1. Supplementary materials

1.1. ANOVA results

Table S1. ANOVA results.

Source of Variation	Variable	Sum of Squares	df	Mean Square	F-ratio	p-value
Corrected Model	R_a	24.868	19	1.309	23.328	0.000
	$\bar{F}_z$	187.642	19	9.876	13,505.383	0.000
	S_z	9.501	19	0.500	5.279	0.000
	Δmax_z	14,175.423	19	746.075	2.470	0.002
	Δmin_z	17,435.193	19	917.642	3.789	0.000
	N_{upp}	3,338,799.092	19	175,726.268	4.063	0.000
	N_{low}	3,286,733.492	19	172,985.973	3.796	0.000
	e_f	12.682	19	0.667	0.865	0.626
Interception	R_a	107.239	1	107.239	1911.380	0.000
	$\bar{F}_z$	1678.053	1	1678.053	2,294,751.409	0.000
	S_z	49.360	1	49.360	521.110	0.000
	Δmax_z	260,026.010	1	260,026.010	860.866	0.000
	Δmin_z	256,687.722	1	256,687.722	1059.932	0.000
	N_{upp}	4,171,259.408	1	4,171,259.408	96.442	0.000
	N_{low}	5,001,675.008	1	5,001,675.008	109.755	0.000
	e_f	12.262	1	12.262	15.891	0.000
Control Type	R_a	0.094	1	0.094	1.677	0.198
	$\bar{F}_z$	0.002	1	0.002	2.951	0.089
	S_z	0.054	1	0.054	0.568	0.453
	Δmax_z	112.937	1	112.937	0.374	0.542
	Δmin_z	234.799	1	234.799	0.970	0.327
	N_{upp}	1491.075	1	1491.075	0.034	0.853
	N_{low}	69.008	1	69.008	0.002	0.969
	e_f	1.927	1	1.927	2.498	0.117
Reference Force	R_a	0.411	1	0.411	7.320	0.008
	$\bar{F}_z$	187.630	1	187.630	256,585.617	0.000

	S_z	4.453	1	4.453	47.012	0.000
	Δmax_z	1004.402	1	1004.402	3.325	0.071
	Δmin_z	949.822	1	949.822	3.922	0.050
	N_{upp}	1,474,305.008	1	1,474,305.008	34.087	0.000
	N_{low}	1,620,990.075	1	1,620,990.075	35.571	0.000
	e_f	1.674	1	1.674	2.169	0.144
Material	R_a	22.432	4	5.608	99.956	0.000
	$\bar{F}_z$	0.006	4	0.001	1.944	0.109
	S_z	2.629	4	0.657	6.938	0.000
	Δmax_z	10,264.652	4	2566.163	8.496	0.000
	Δmin_z	9325.331	4	2331.333	9.627	0.000
	N_{upp}	932,522.883	4	233,130.721	5.390	0.001
	N_{low}	922,109.617	4	230,527.404	5.059	0.001
	e_f	4.102	4	1.025	1.329	0.264
Control Type * Reference Force	R_a	0.010	1	0.010	0.180	0.673
	$\bar{F}_z$	$2.828 \cdot 10^{-7}$	1	$2.828 \cdot 10^{-7}$	0.000	0.984
	S_z	0.172	1	0.172	1.814	0.181
	Δmax_z	397.822	1	397.822	1.317	0.254
	Δmin_z	171.097	1	171.097	0.707	0.403
	N_{upp}	90,036.408	1	90,036.408	2.082	0.152
	N_{low}	21,253.408	1	21,253.408	0.466	0.496
	e_f	0.201	1	0.201	0.261	0.611
Control Type * Material	R_a	0.504	4	0.126	2.244	0.070
	$\bar{F}_z$	0.001	4	0.000	0.397	0.810
	S_z	0.390	4	0.098	1.030	0.396
	Δmax_z	98.097	4	24.524	0.081	0.988
	Δmin_z	731.837	4	182.959	0.755	0.557
	N_{upp}	79,586.883	4	19,896.721	0.460	0.765
	N_{low}	138,840.950	4	34,710.238	0.762	0.553
	e_f	1.807	4	0.452	0.585	0.674
Reference Force * Material	R_a	0.824	4	0.206	3.673	0.008
	$\bar{F}_z$	0.002	4	0.000	0.616	0.652
	S_z	1.434	4	0.359	3.785	0.007
	Δmax_z	1613.502	4	403.375	1.335	0.262
	Δmin_z	5328.349	4	1332.087	5.501	0.000
	N_{upp}	510,518.617	4	127,629.654	2.951	0.024
	N_{low}	495,118.383	4	123,779.596	2.716	0.034
	e_f	0.995	4	0.249	0.322	0.862
Control Type * Reference Force * Material	R_a	0.593	4	0.148	2.642	0.038
	$\bar{F}_z$	0.001	4	0.000	0.469	0.758
	S_z	0.369	4	0.092	0.975	0.425
	Δmax_z	684.011	4	171.003	0.566	0.688
	Δmin_z	693.957	4	173.489	0.716	0.583
	N_{upp}	250,338.217	4	62,584.554	1.447	0.224
	N_{low}	88,352.050	4	22,088.013	0.485	0.747
	e_f	1.975	4	0.494	0.640	0.635
Error	R_a	5.611	100	0.056		

	$\bar{F}_z$	0.073	100	0.001
	S_z	9.472	100	0.095
	Δmax_z	30,205.164	100	302.052
	Δmin_z	24,217.371	100	242.174
	N_{upp}	4,325,158.500	100	43,251.585
	N_{low}	4,557,116.500	100	45,571.165
	e_f	77.166	100	0.772
Total	R_a	137.717	120	
	$\bar{F}_z$	1865.769	120	
	S_z	68.332	120	
	Δmax_z	304,406.597	120	
	Δmin_z	298,340.286	120	
	N_{upp}	11,835,217.000	120	
	N_{low}	12,845,525.000	120	
	e_f	102.110	120	
Corrected Total	R_a	30.478	119	
	$\bar{F}_z$	187.715	119	
	S_z	18.973	119	
	Δmax_z	44,380.587	119	
	Δmin_z	41,652.564	119	
	N_{upp}	7,663,957.592	119	
	N_{low}	7,843,849.992	119	
	e_f	89.848	119	

1.2. Sanding in aluminium

1.2.1. Experiment E1

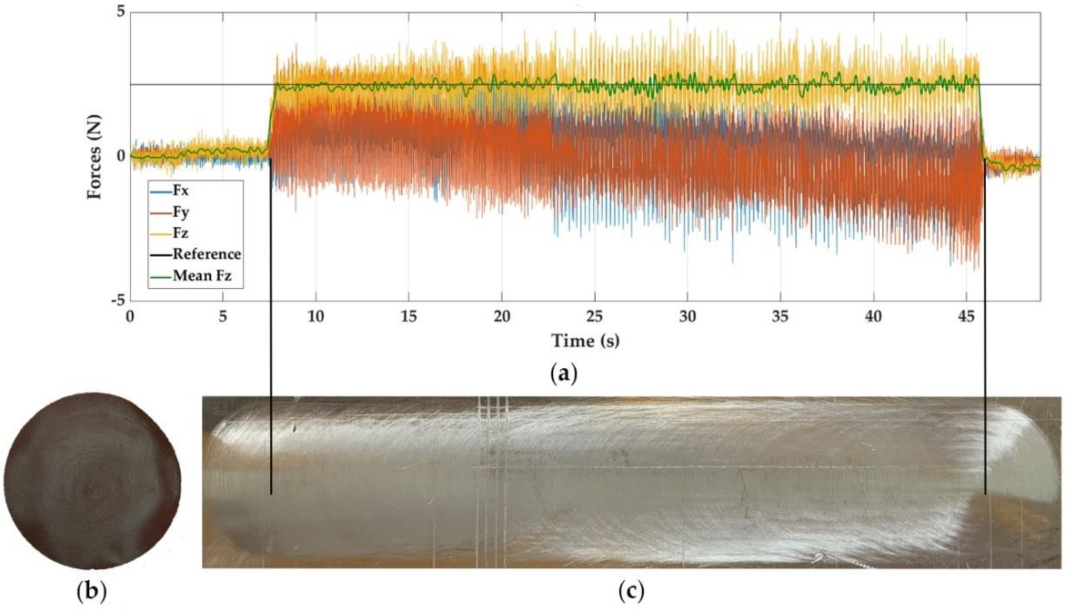

Figure S1. Experiment E1, sanding aluminium with P+FF control and reference force of 2.5 N. (**a**) Force response, (**b**) sandpaper and (**c**) surface finish.

1.2.2. Experiment E3

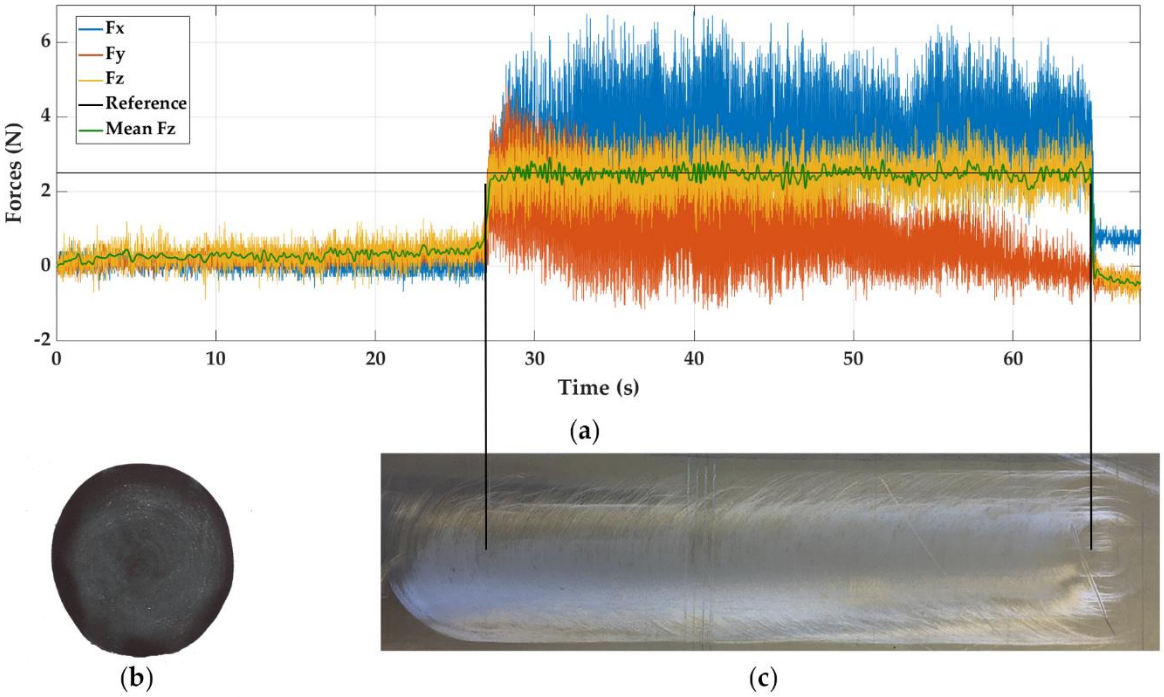

Figure S2. Experiment E3, sanding aluminium with PIV control and reference force of 2.5 N. (**a**) Force response, (**b**) sandpaper and (**c**) surface finish.

1.2.3. Experiment E4

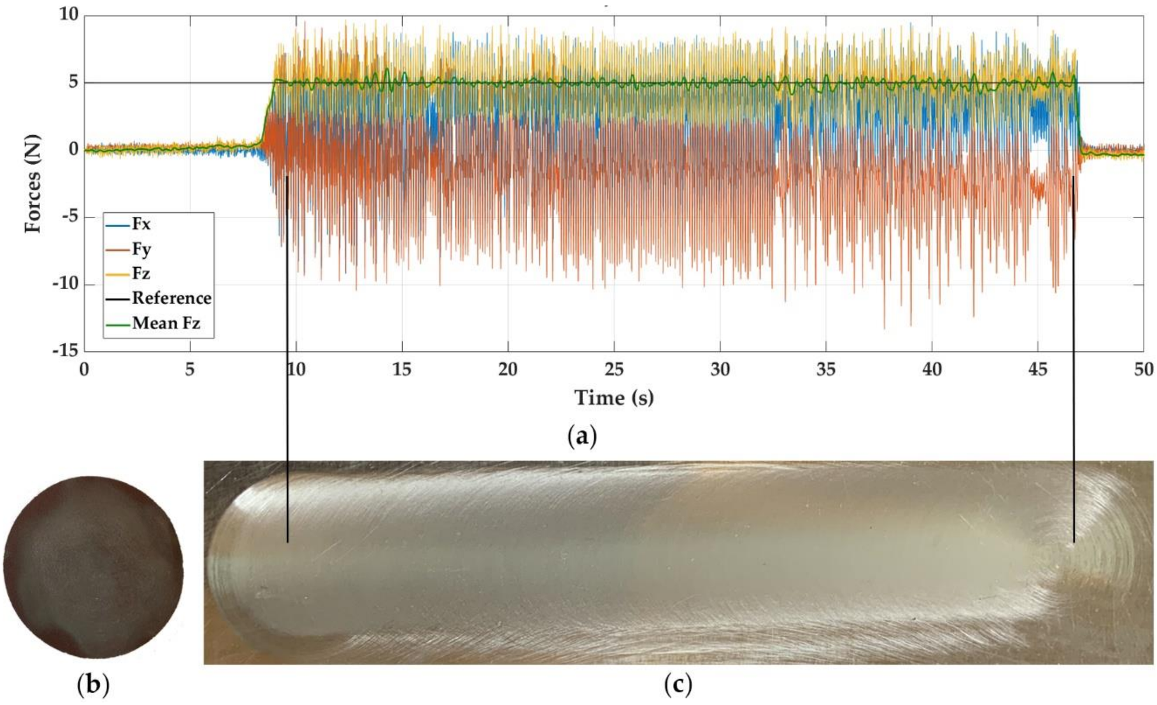

Figure S3. Experiment E4, sanding aluminium with PIV control and reference force of 5 N. (**a**) Force response, (**b**) sandpaper and (**c**) surface finish.

1.3. Sanding in steel

1.3.1. Experiment E5

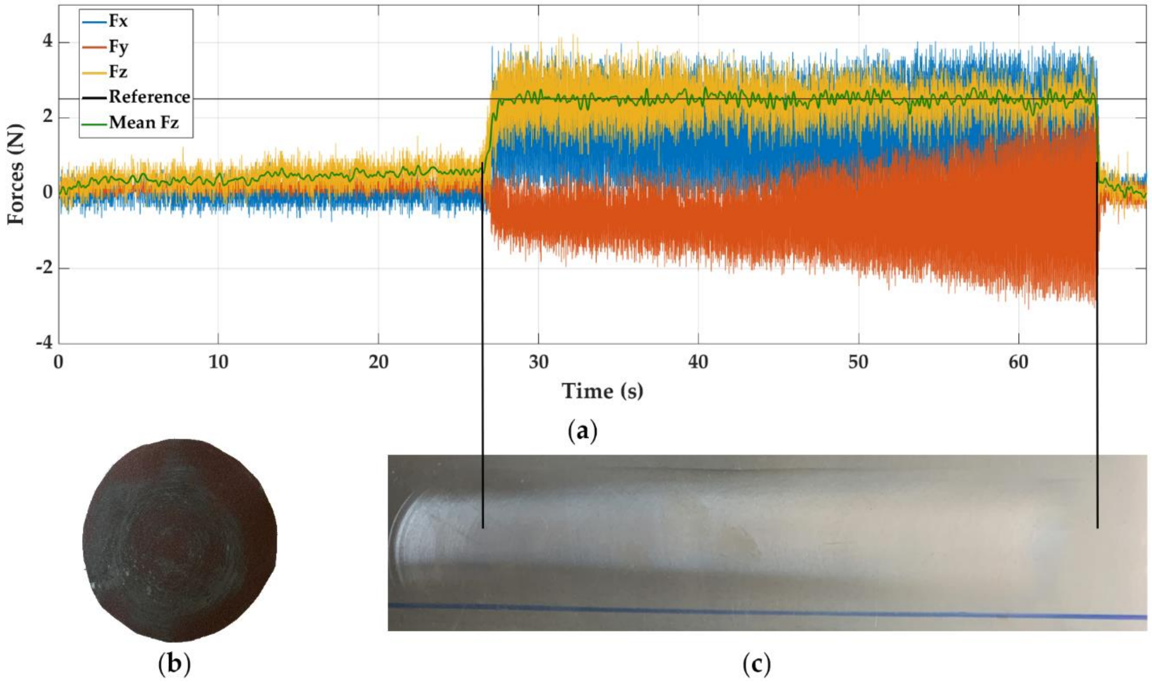

Figure S4. Experiment E5, sanding steel with P+FF control and reference force of 2.5 N. (**a**) Force response, (**b**) sandpaper and (**c**) surface finish.

1.3.2. Experiment E7

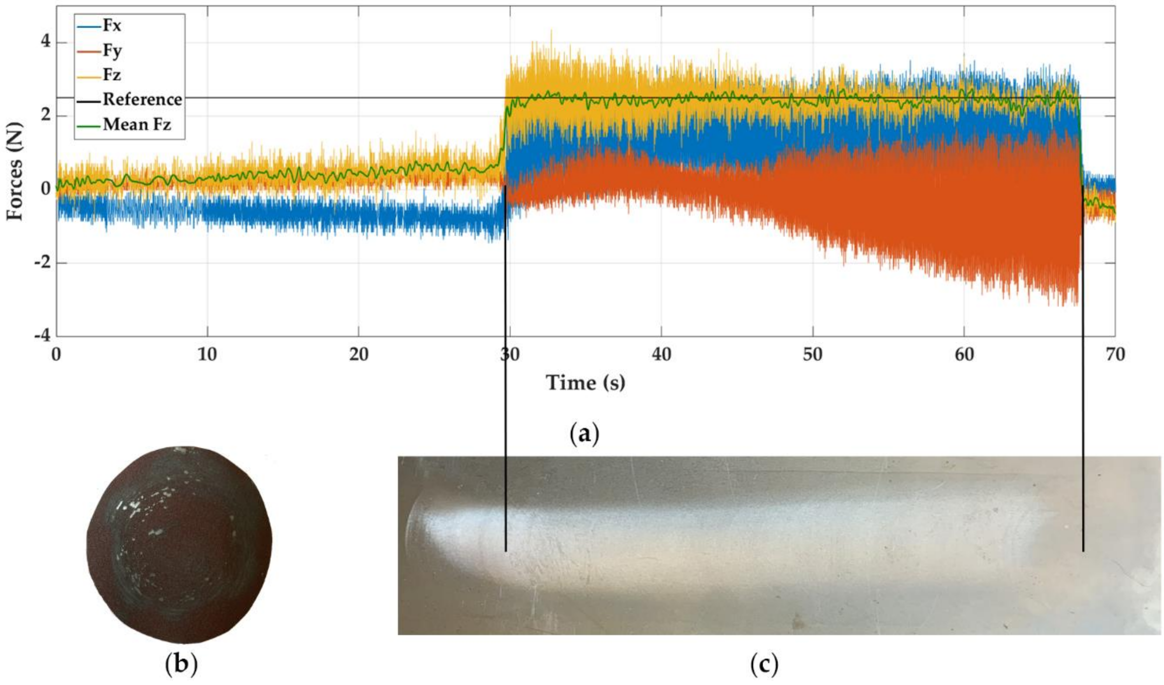

Figure S5. Experiment E7, sanding steel with PIV control and reference force of 2.5 N. (**a**) Force response, (**b**) sandpaper and (**c**) surface finish.

1.3.3. Experiment E8

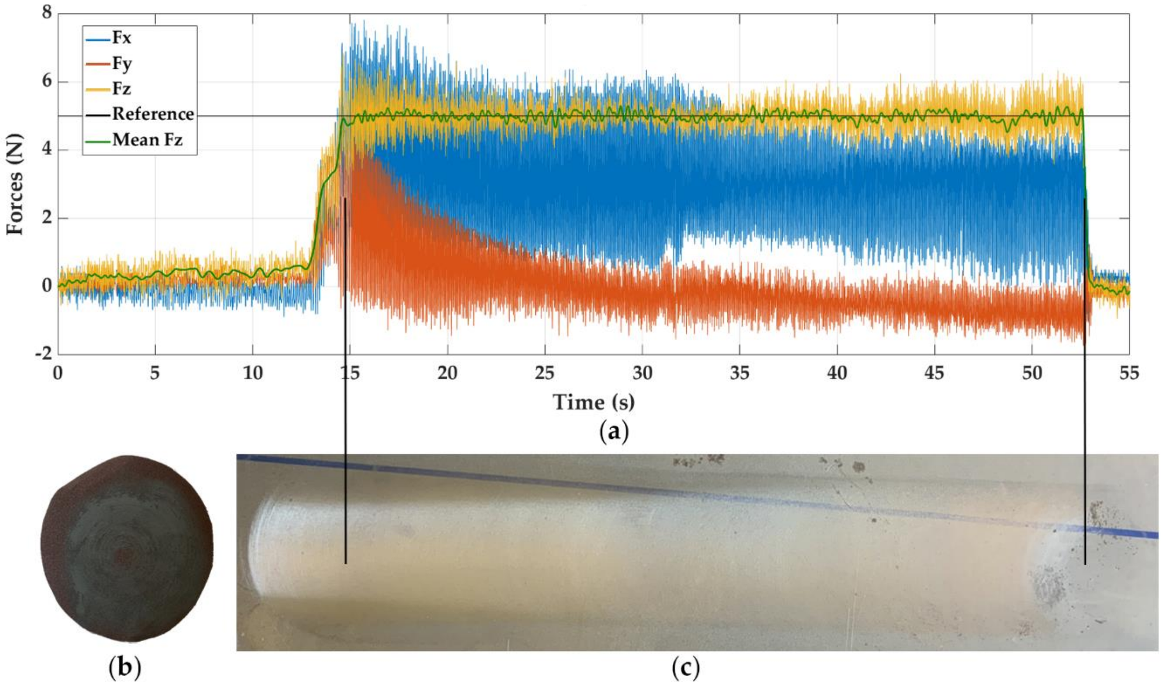

Figure S6. Experiment E8, sanding steel with PIV control and reference force of 5 N. (**a**) Force response, (**b**) sandpaper and (**c**) surface finish.

1.4. Sanding in brass

1.4.1. Experiment E9

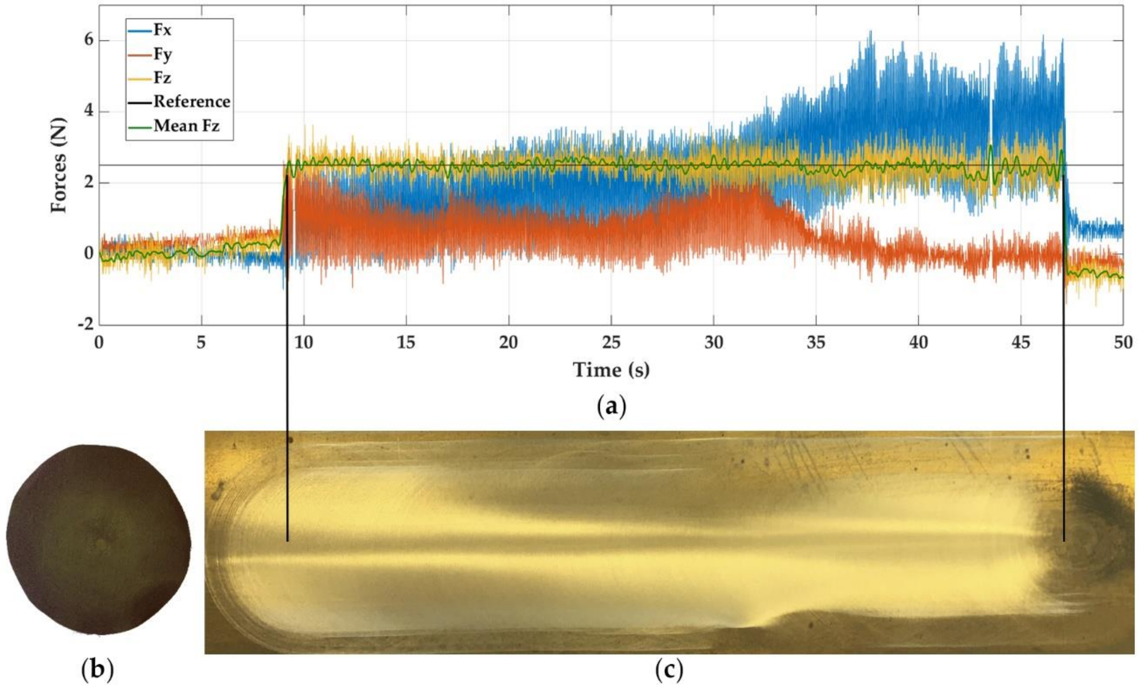

Figure S7. Experiment E9, sanding brass with P+FF control and reference force of 2.5 N. (**a**) Force response, (**b**) sandpaper and (**c**) surface finish.

1.4.2. Experiment E10

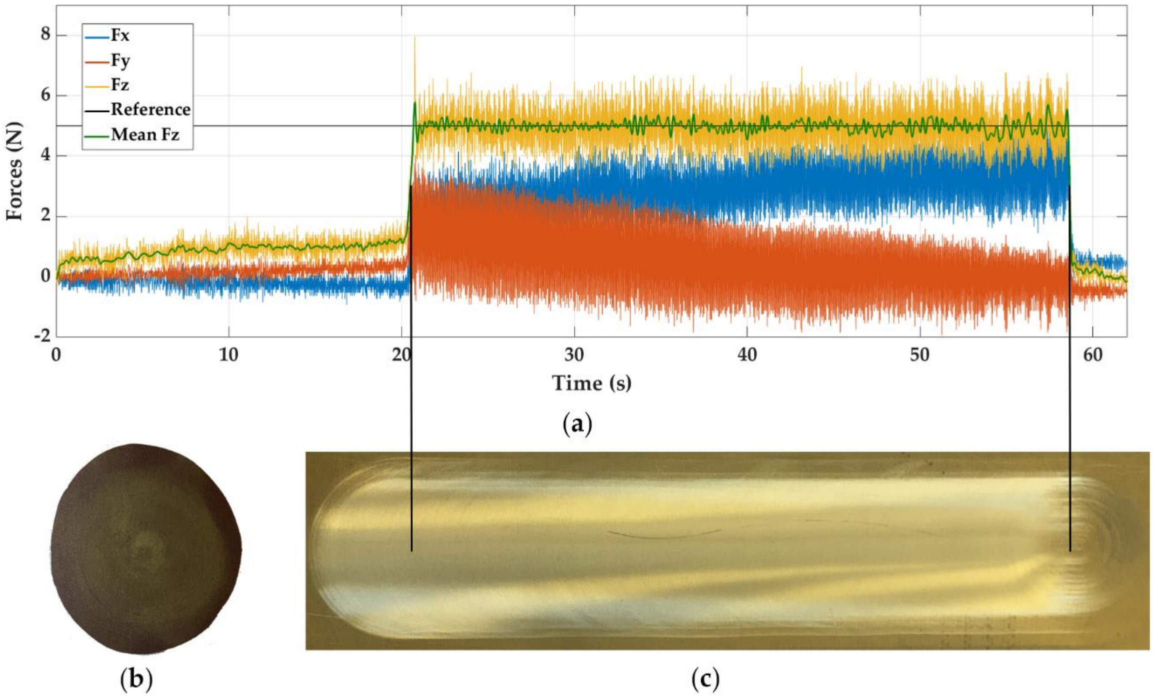

Figure S8. Experiment E10, sanding brass with P+FF control and reference force of 5 N. (**a**) Force response, (**b**) sandpaper and (**c**) surface finish.

1.4.3. Experiment E11

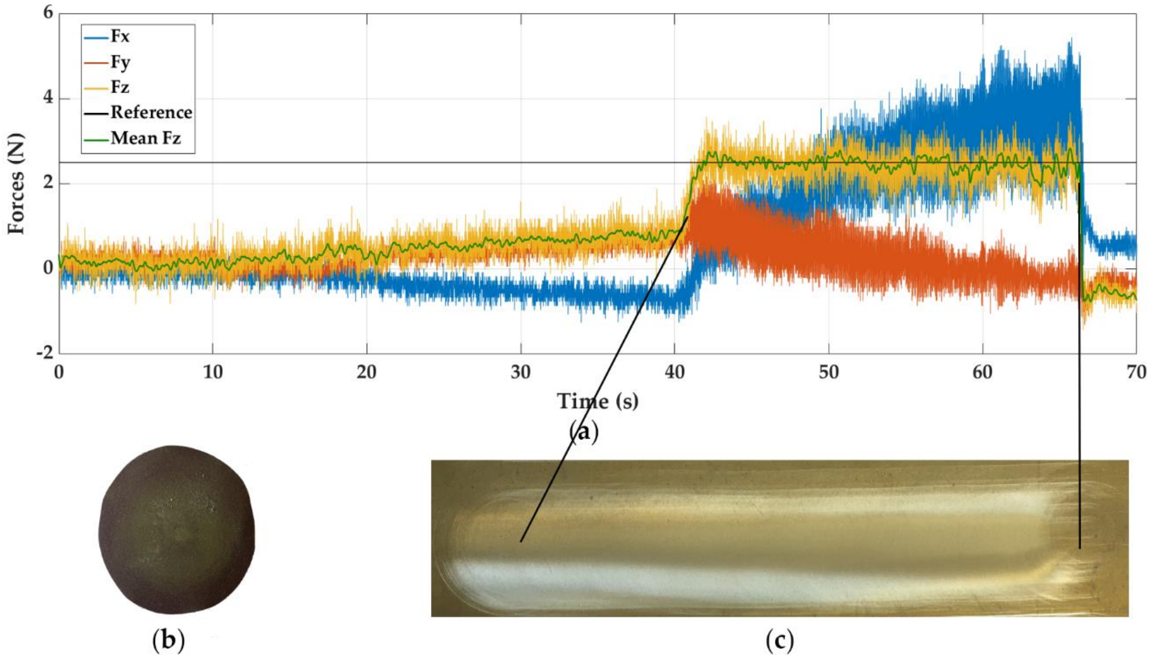

Figure S9. Experiment E11, sanding brass with PIV control and reference force of 2.5 N. (**a**) Force response, (**b**) sandpaper and (**c**) surface finish.

1.5. Sanding in wood

1.5.1. Experiment E13

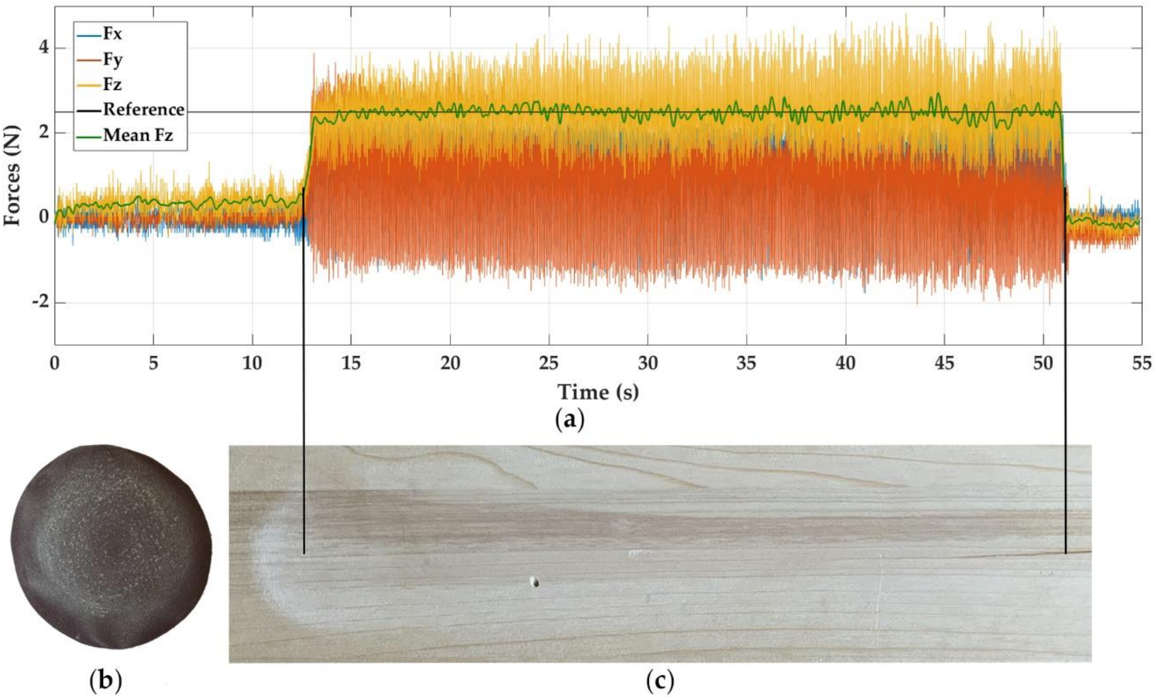

Figure S10. Experiment E13, sanding wood with P+FF control and reference force of 2.5 N. (**a**) Force response, (**b**) sandpaper and (**c**) surface finish.

1.5.2. Experiment E15

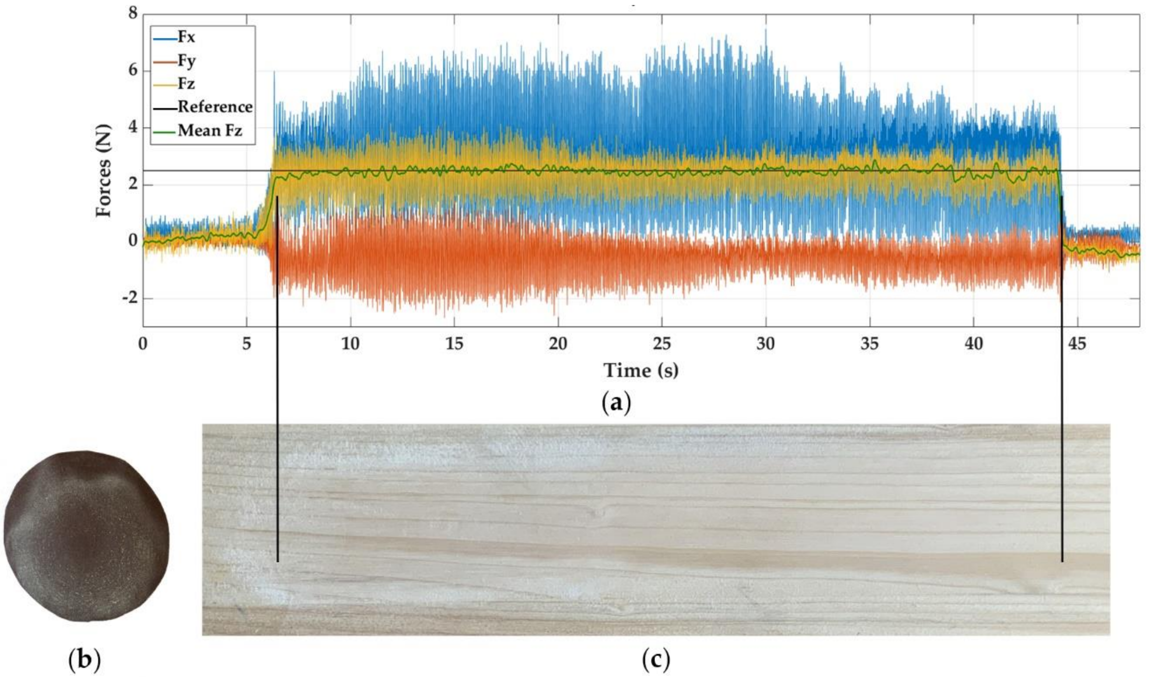

Figure S11. Experiment E15, sanding wood with PIV control and reference force of 2.5 N. (**a**) Force response, (**b**) sandpaper and (**c**) surface finish.

1.5.3. Experiment E16

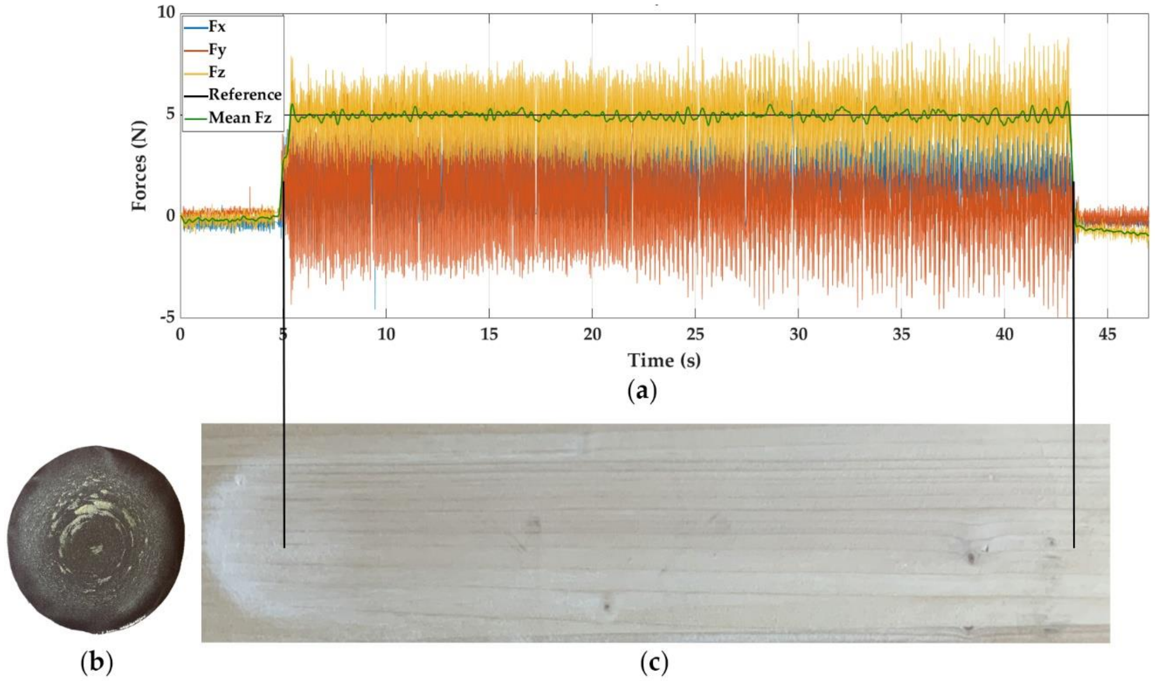

Figure S12. Experiment E16, sanding wood with PIV control and reference force of 5 N. (**a**) Force response, (**b**) sandpaper and (**c**) surface finish.

1.6. Sanding in PVC

1.6.1. Experiment E17

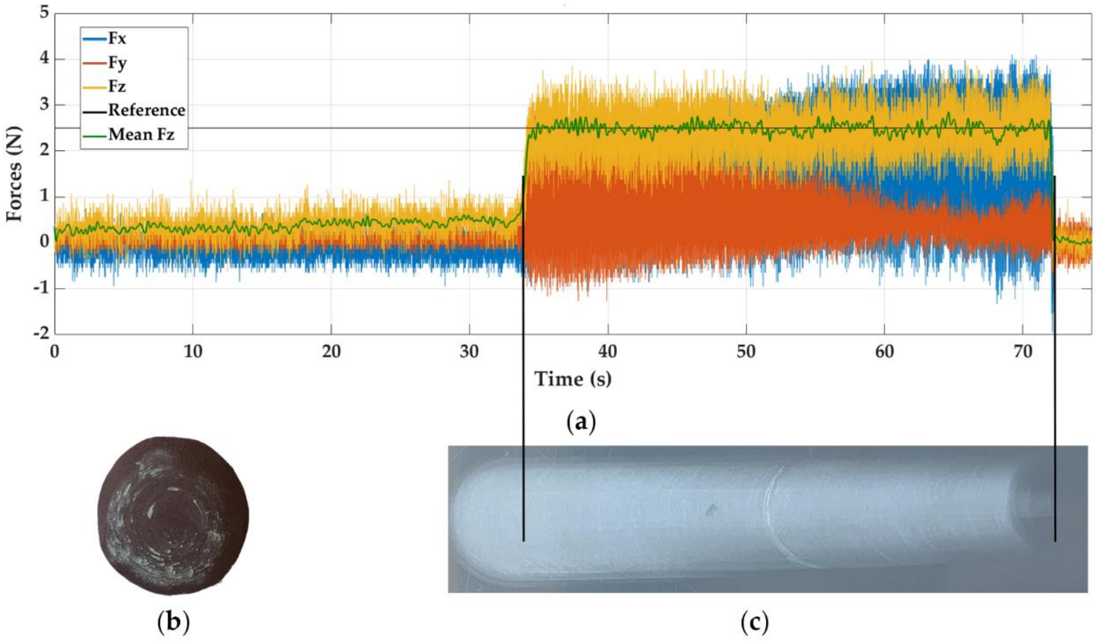

Figure S13. Experiment E17, sanding PVC with P+FF control and reference force of 2.5 N. (**a**) Force response, (**b**) sandpaper and (**c**) surface finish.

1.6.2. Experiment E18

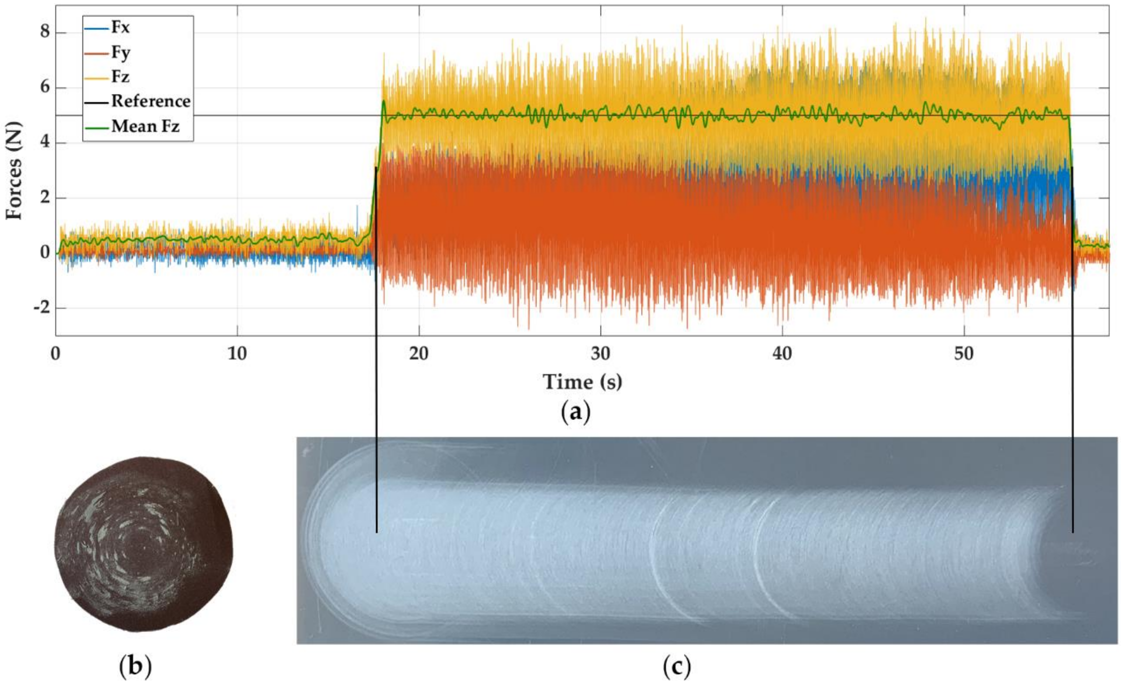

Figure S14. Experiment E18, sanding PVC with P+FF control and reference force of 5 N. (**a**) Force response, (**b**) sandpaper and (**c**) surface finish.

1.6.3. Experiment E19

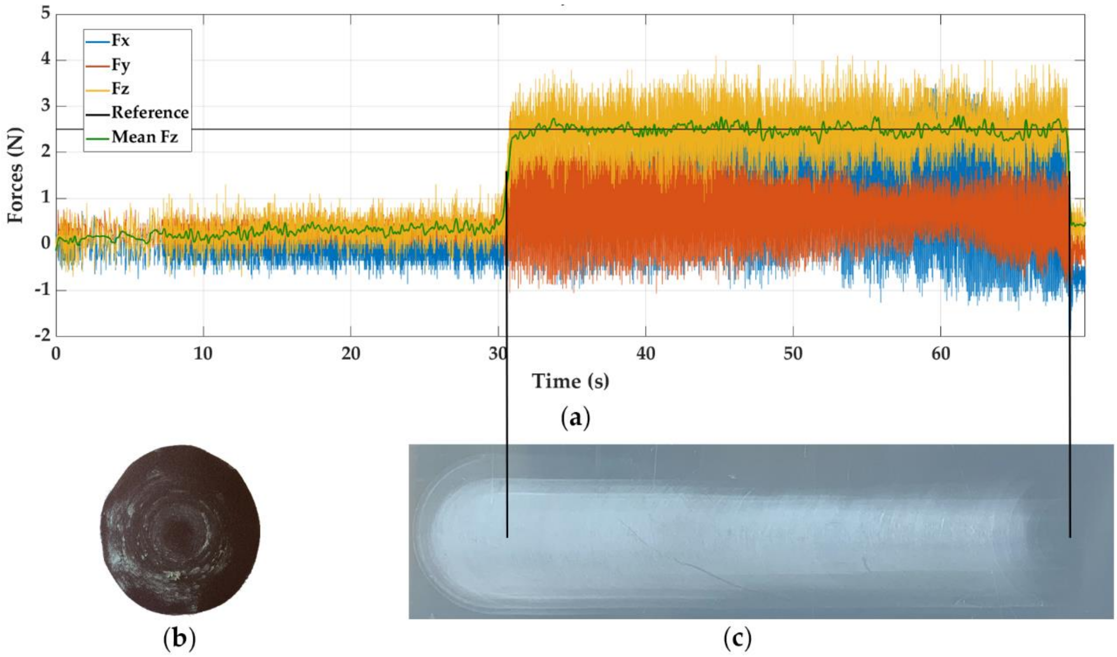

Figure S15. Experiment E19, sanding PVC with PIV control and reference force of 2.5 N. (**a**) Force response, (**b**) sandpaper and (**c**) surface finish.
